# MicroRNA 19a replacement partially rescues fin and cardiac defects in zebrafish model of Holt Oram syndrome

**DOI:** 10.1038/srep18240

**Published:** 2015-12-14

**Authors:** Elena Chiavacci, Romina D’Aurizio, Elena Guzzolino, Francesco Russo, Mario Baumgart, Marco Groth, Laura Mariani, Mara D’Onofrio, Ivan Arisi, Marco Pellegrini, Alessandro Cellerino, Federico Cremisi, Letizia Pitto

**Affiliations:** 1 Institute of Clinical Physiology, National Research Council, via Moruzzi 1, 56124 Pisa, Italy; 2 Laboratory of Integrative Systems Medicine (LISM), Institute of Informatics andTelematics (IIT) and Institute of Clinical Physiology (IFC), (CNR), Pisa, Italy; 3 Institute of Life Sciences, Scuola Superiore Sant’Anna, Piazza Martiri della Libertà 33, 56127 Pisa, Italy; 4 Department of Computer Science, University of Pisa, Pisa, Italy; 5 Leibniz Institute for Age Research - Fritz Lipmann Institute (FLI), Beutenbergstr. 11, 07745 Jena, Germany; 6 Genomics Facility, European Brain Research Institute, Via del Fosso di Fiorano 64 00143 Roma, Italy; 7 Scuola Normale Superiore di Pisa, Piazza dei Cavalieri 7, 56100 Pisa, Italy

## Abstract

Holt-Oram Syndrome (HOS) is an autosomal dominant heart-hand syndrome caused by mutations in the *TBX5* gene, a transcription factor capable of regulating hundreds of cardiac-specific genes through complex transcriptional networks. Here we show that, in zebrafish, modulation of a single miRNA is sufficient to rescue the morphogenetic defects generated by HOS. The analysis of miRNA-seq profiling revealed a decreased expression of miR-19a in Tbx5-depleted zebrafish embryos compared to the wild type. We revealed that the transcription of the miR-17-92 cluster, which harbors miR-19a, is induced by Tbx5 and that a defined dosage of miR-19a is essential for the correct development of the heart. Importantly, we highlighted that miR-19a replacement is able to rescue cardiac and pectoral fin defects and to increase the viability of HOS zebrafish embryos. We further observed that miR-19a replacement shifts the global gene expression profile of HOS-like zebrafish embryos towards the wild type condition, confirming the ability of miR-19a to rescue the Tbx5 phenotype. In conclusion our data demonstrate the importance of Tbx5/miR-19a regulatory circuit in heart development and provide a proof of principle that morphogenetic defects associated with HOS can be rescued by transient miRNA modulation.

Micro-RNAs (miRNAs) are evolutionarily-conserved noncoding RNAs approximately 22 nucleotides in length, which negatively regulate gene expression by translational repression or mRNA destabilization^1^,^2^. miRNAs have been implicated in numerous diseases^3^,^4^,^5^, including cancer^6^,^7^. The hypothesis that miRNA dysregulation is part of pathophysiological mechanisms underlying heart disease was first suggested by distinctive patterns of miRNA expression discovered in healthy and diseased mouse and human hearts (reviewed in^8^,^9^,^10^). miRNAs are now demonstrated to be actively involved in all aspects of cardiac remodeling, growth, proliferation, apoptosis, conductance and contractility. Thanks to their small size, their conserved and well characterized sequences, and their ability to impact multiple mRNAs, which are often functionally related, miRNAs are able to modulate complex physiological phenotypes by fine-tuning entire functional networks and therefore are attractive potential targets for complex disease therapy^11^,^12^. Holt Oram syndrome (HOS) is an autosomal dominant disorder characterized by cardiac and upper limb abnormalities^13^. Mutations in T-box transcription factor 5 (*TBX5*) underlie this syndrome^14^,^15^. Even small perturbations in *TBX5* expression have significant effects on the HOS phenotype and modify the expression of hundreds of genes as shown in a murine model of HOS^16^,^17^. Interestingly, although TBX5 has been exclusively characterized as transcriptional activator, about 30% of the genes identified by microarray as being differentially expressed in *tbx5* haploinsufficient mice are upregulated. This result suggests that TBX5 exerts its action at least in part via indirect mechanisms, such as, for example, activation of repressors. Our working hypothesis is that miRNAs are critical negative effectors of TBX5: more specifically the peculiar ability of each miRNA to modulate many targets might contribute to expand the range of influence of TBX5 which is a pivotal gene in heart morphogenesis. In line with our hypothesis, we identified in zebrafish HOS model those miRNAs embedded in genes highly sensitive to *Tbx5* dosage. In our previous work we showed that misregulation of miR-218, has a severe impact on heart development by affecting early heart morphogenesis^18^.

In the present work we first performed massive parallel sequencing of the small RNAs in zebrafish embryos depleted for Tbx5 by morpholino injection (Tbx5-morphants). Then, by analysing the RNA profiles, we identified the miRNAs downregulated in Tbx5-morphants. Among several differentially regulated miRNAs we selected miR-19a because of its capability to significantly rescue the cardiac and pectoral fin defects caused by Tbx5 depletion and because of its capability to improve the Tbx5 morphant viability when co-injected with the morpholino against Tbx5. By *in situ* hybridization, we demonstrated that the proper expression of crucial cardiac genes is restored by miR-19a replacement. Moreover we showed, by microarray analysis, that gene expression profile of Tbx5 morphants co-injected with miR-19a mimic clusters together with WT embryos. Furthermore several miR-19 targets, some of them with relevance for cardiovascular development, are found to be up regulated by Tbx5 depletion and restored to WT condition by miR-19a co-injection.

## Results

### Tbx5a/b downregulation misregulates miRNA expression during zebrafish development

**Figure 1:**
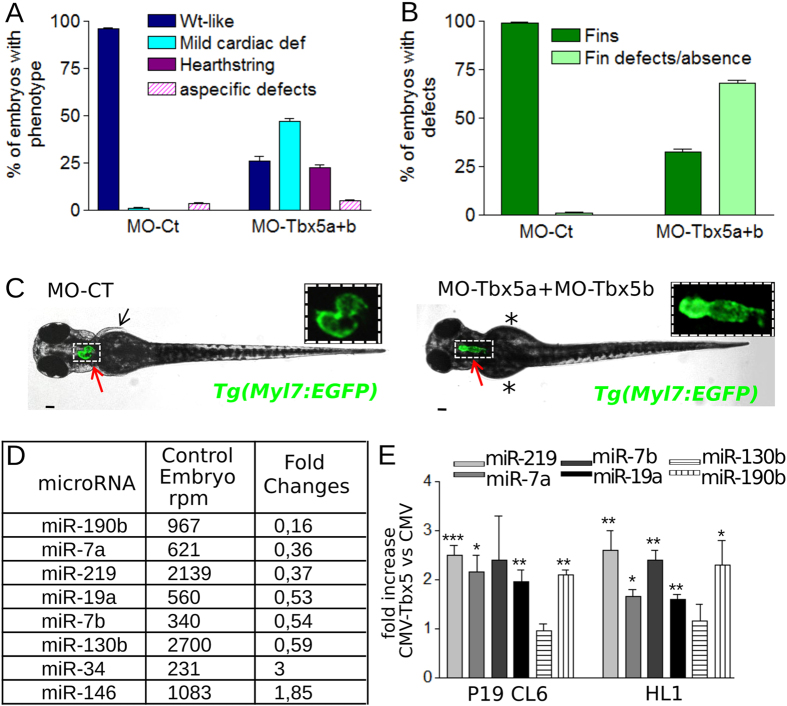
Tbx5a/b downregulation dysregulates miRNA expression during zebrafish development. (**A,B**) Cardiac (**A**) and fin (**B**) analysis of 72 hpf embryos injected with 3 ng of MO-Ct or 1.5 ng of MO-Tbx5a and 1.5 ng of MO-Tbx5b. (**C**) Images representative of WT-like and heartstring *Tg(Myl7:EGFP)* morphants at 72 hpf. Red and black arrows point to heart and fins, respectively. Stars mark fin absence. Scale bar 100 μm. Heart higher magnification in insets. (**D**) List of miRNAs that showed a positive or negative fold change ≥1.8 and a number of reads per million (rpm) higher than 200 in the Tbx5 depleted embryos (MO-Tbx5a + MO-Tbx5b). (**E**) QRT-PCR analysis of mouse P19CL6 proliferating cells or HL1 cells 48 hrs after transfection with Tbx5 expressing vector or with an empty vector. t-test was used for statistical analysis.

In order to identify miRNAs modulated by Tbx5, we simultaneously depleted both zebrafish Tbx5 paralogs^19^,^20^ by microinjecting embryos at 1-cell stage with morpholinos against Tbx5a and Tbx5b (MO-Tbx5a and MO-Tbx5b). These morpholinos have been already extensively used to functionally analyze Tbx5 paralogs^19^,^20^,^21^,^22^,^23^. About 70% of embryos injected with 1.5ng of MO-Tbx5a and 1.5 ng of MO-Tbx5b showed cardiac and fin defects (Fig. 1A,B). Embryos showing the typical heartstring (hts) phenotype associated to Tbx5 downregulation^18^,^23^ were manually selected at 48hpf under microscope (Fig. 1C). Total RNA was extracted from Tbx5 depleted embryos and from embryos microinjected with the same amount of control morpholino (MO-Ct)^18^. miRNA profiles were performed by next generation sequencing (NGS). We identified 8 miRNAs with a fold change (FC) higher than 1.8 and a number of reads per million (rpm) higher than 200 of which 6 down- and 2 up-regulated in hts morphants compared to MO-Ct injected embryos (Fig. 1D). Next, we focused our attention on miRNAs showing down-regulation as a consequence of Tbx5 depletion, which is the premise for a Tbx5 direct regulation. To test whether Tbx5 can induce the expression of these miRNAs, we over-expressed Tbx5 in two different mouse cardiac cell lines, P19CL6^24^,^25^ and HL1 cells^26^. We showed by qRT-PCR analysis that the expression levels of miR-219, miR-190b, miR-19a, miR-7a and miR-7b significantly increase as a consequence of Tbx5 overexpression (Fig. 1E).

### miR-19a is able to partially rescue heart/fin defects induced by MO-Tbx5

**Figure 2:**
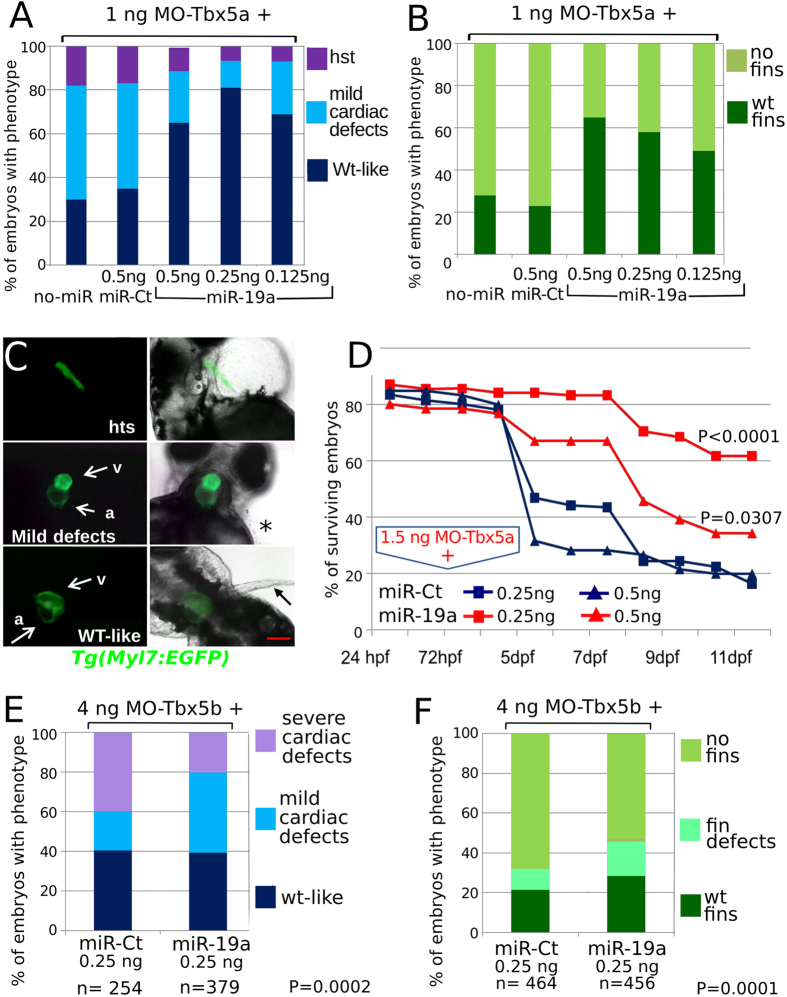
miR-19a mimic is able to partially rescue heart/fin defects induced by MO-Tbx5. (**A–C**) Analysis of 72hpf Tbx5a morphants. miR-19a or miR-Ct mimics, at the reported doses, were co-injected with 1.5 ng of MO-Tbx5a in *Tg(Myl7:EGFP)* embryos. The percentage of embryos with the indicated heart (**A**) or pectoral fin (**B**) defects was averaged across multiple independent experiments carried out in double blind. The total number of analyzed Tbx5 morphant embryos were as follows: not co-injected n = 100; miR-Ct co-injected n = 272 (0.5 ng), n = 214 (0.25 ng), n = 164 (0.125ng); miR-19a co-injected n = 200 (0.5ng), n = 132 (0.25 ng), n = 260 (0.125 ng). For each miR-19a mimic dose, the equivalent dose of miR-Ct was always injected as control. For all tested doses, differences between miR-Ct and miR-19a were significant (Fisher’s test P < 0,0001). In (**C**) images representative of the different phenotypes. Red scale bar = 10 μm, hts, heartstring; v, ventricle; a, atrium. White arrows indicate cardiac chambers, black arrow and star indicate fin presence and absence respectively. (**D**) Embryos injected with the indicated mix of morpholino and miRNA mimics were followed for 11 days, daily recording the number of surviving embryos. Curves were compared by long-rank test analysis. The total number of analyzed embryos were as follows: miR-Ct co-injected n = 60 (0.5 ng), n = 116 (0,25 ng); miR-19a co-injected n = 71 (0,5 ng), n = 97 (0,25 ng). (**E,F**) Analysis of 72hpf Tbx5b morphants co-injected with 4 ng of Tbx5b morpholino and 0.25 ng of miR-19a or miR-Ct mimics. hts: heartstring phenotype; mild defects: general cardiac defects; WT: wild type heart. NO fins: pectoral fins absence, WT fins: wild type pectoral fins.

We investigated whether miR-219, miR-190b, miR-19a, 130a and miR-7b could be effectors of Tbx5 activity by testing whether the replacement of any of these miRNAs alone was able to rescue morphological defects induced by Tbx5 depletion^18^,^23^. The tool used for this screening is the transgenic *Tg(Myl7:EGFP)* zebrafish line showing EGFP-labelled cardiomyocytes. Since we had already shown that the penetrance of the MO-Tbx5b phenotype was lower compared to that of MO-Tbx5a^18^, we used only MO-Tbx5a for all the subsequent Tbx5 depletion experiments. *Tg(Myl7:EGFP)* embryos were injected with 1.5 ng of MO-Tbx5a and 0.25 ng of each miRNA mimic. Co-injection of miR-130a and miR-219 was ineffective while co-injection of miR-7b and miR-190b even worsened the Tbx5a morphant phenotypes (Fig. S1). On the other hand, doses below 1 ng of miR-19a mimic significantly reduce both cardiac and fin defects (Fig. 2A–C). Co-injection of MO-Tbx5a and non-coding miRNA mimic (miR-Ct) does not improve morphant phenotype (Fig. 2A–C).

We questioned whether the injection of miR-19a would improve the viability of Tbx5a morphants. Figure 2D shows that about 60% of the Tbx5a morphants co-injected with 0.25/0.5 ng of miR-Ct die around the 5^th^–6^th^ day of development, when the oxygen exchange by passive diffusion is no longer sufficient to sustain aerobic metabolism and a functioning circulatory system becomes necessary for survival. Less than 20% of Tbx5a morphants were still viable at the 11^th^ day of development. Conversely, more than 60% of morphants co-injected with 0.25 ng of miR-19a mimic were still alive at the 12^th^ day of development (Fig. 2D). In line with the dose-dependency of the cardiac rescue data (Fig. 2A,B), co-injection of 0.5 ng of miR-19a was less effective than a lower dose in improving viability. To explain this result we speculate that a specific dosage of miR-19a would be necessary for proper heart development. Moreover, since miRNAs are fine tuned rheostatic regulators of biological processes by targeting many mRNAs, an excessive amount of a single miRNAs can lead to nonspecific repression of unrelated mRNAs, causing a worsening of the phenotype.

Replacement of miR-19a decreased the severity of cardiac defects produced by Tbx5b depletion too, although it did not increase the number of normal embryos (Fig. 2E). This result is in accordance with the partial redundancy of zebrafish Tbx5 paralog functions^20^.

### Tbx5 modulation affects miR-17-92 cluster expression

**Figure 3:**
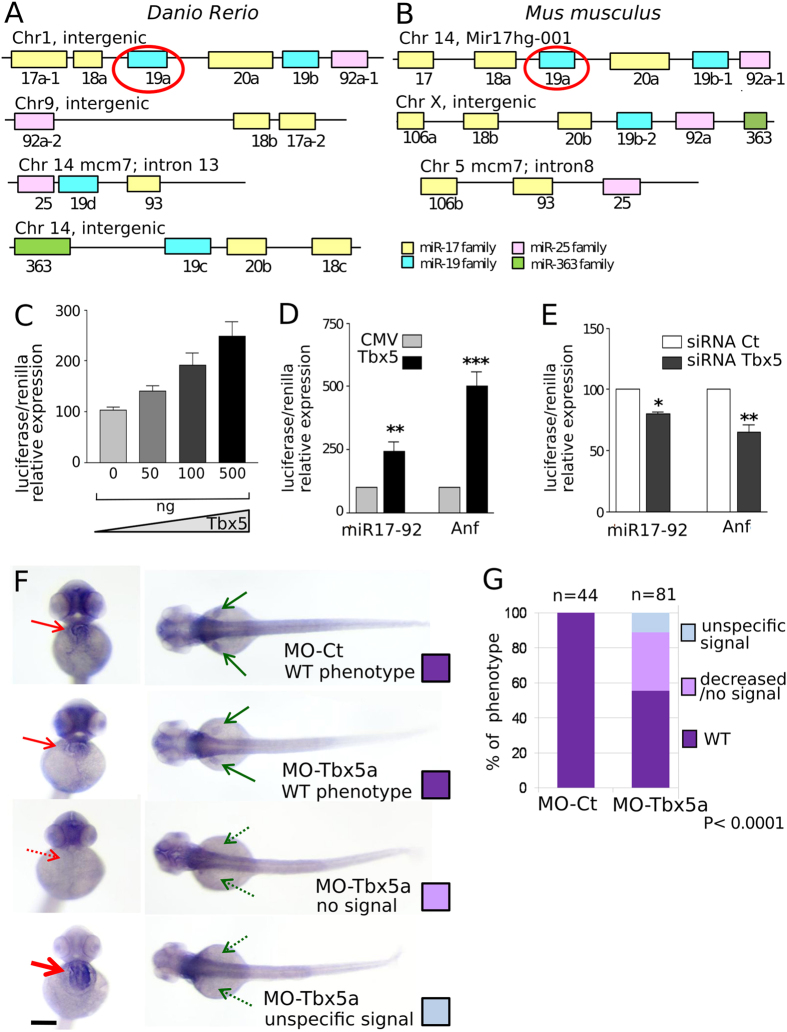
Tbx5 modulation affects miR-17-92 cluster expression. (**A,B**) Schematic representation of miR-17-92 paralogs in zebrafish and mouse. The color code identifies miRNAs of the same family. (**C**) Relative Luciferase/Renilla activity in HL1 cells transfected with pGL3-MIR (containing a fragment of 1600 bp of miR-17-92 cluster promoter upstream of the luciferase reporter gene) and the pRL-TK renilla vector in the presence of increasing doses (0–500 ng) of pCMV-Tbx5 vector; (**D,E**) Relative Luciferase/Renilla activity in HL1 cells transfected with 500 ng of pGL3-MIR or 500 ng of pGL3 vector containing the Anf promoter (Anf) in the presence of (**D**) 500 ng of CMV-Tbx5 or the empty CMV vector; (**E**) Tbx5 specific siRNA (siRNA-Tbx5) or nonspecific siRNA (siRNA Ct). The y-axis represents fold activation of reporter normalized by a dual-luciferase system. t-test was used for statistical analysis *P < 0.05. (**F**) miR-17-92 cluster ISH on 72 hpf embryos injected with 1.5 ng of MO-Ct or 1.5 ng of MO-Tbx5a. Examples of the different phenotypes observed in the Tbx5 morphants are shown. Red and green arrows indicate heart and fins, respectively. Dashed arrows highlight the absence of expression, thicker arrow indicates over-expression. Scale bar 100 μm. (**G**) Quantification of the different phenotypes as in (**F**). A color code is used to identify the different phenotypes: only the WT (purple) phenotype is present in embryos injected with MO-Ct (upper panel of 3F), while 3 different phenotypes are recognizable in embryos injected with MO-Tbx5a. Fisher’s test P value is reported. n, numbers of embryos analyzed.

We investigated whether Tbx5 directly regulates the expression of the miR-17-92 cluster, to which miR-19a belongs. The functional analysis of miR-17-92 members is complicated in vertebrates by the existence of several paralogs (Fig. 3A,B). However, there is only one miRNA-19a in fish as well as in human and rodent genomes^27^.

We scanned the genomic sequence upstream the microRNA cluster for Tbx5 consensus binding sites by TRANSFAC program^28^. A potential Tbx5 binding site was identified about 4500 bp upstream the putative transcription starting site of the miR-17-92 cluster. A sequence of 1600 bp spanning the Tbx5 binding site was cloned into the pGL3 promoter vector upstream of the luciferase reporter gene generating the pGL3-MIR construct. Transactivation assays were performed in HL1 cells transfected with different amounts of the Tbx5 expression vector and with pGL3-MIR in the presence of the pRL-TK vector as internal standard. Co-transfection of increasing doses of the pcDNA-Tbx5 resulted in a progressive increase of luciferase activity (Fig. 3C). Next, the transactivating ability of Tbx5 on the pGL3-MIR vector was compared with the capability of Tbx5 to transactivate the pGL3-Anf construct, which expresses luciferase under the control of the atrial natriuretic factor (Anf) promoter, an established direct target of Tbx5^16^. A dose of 500 ng of the Tbx5 plasmid increases the activity of the pGL3-MIR and pGL3-Anf vectors by 2.5- and 5-folds respectively (Fig. 3D). In line with this data, transfection of a siRNA able to almost halve endogenous Tbx5 expression decreased the luciferase activity both in pGL3-MIR and pGL3-Anf (Fig. 3E).

Downregulation of the miR-17-92 cluster expression in Tbx5a morphants was further supported by whole mount *In Situ* Hybridization (ISH) experiments using a riboprobe for the primary transcript of the miR-17-92 cluster. We observed that more than 30% of the MO-Tbx5a injected embryos have reduced miR-17-92 expression in the cardiac district (Fig. 3F,G). Since the first consequence of Tbx5a depletion was the lack of pectoral fins, we could not evaluate miR-17-92 cluster modulation in this district. Embryos with reduced miR-17-92 hybridization signal always showed a strong phenotype and absence of pectoral fins. More specifically, pectoral fins, whose presence is suggestive of WT-like phenotype (20% of Tbx5a-morphants, Figs S1 and S2), never showed visible reduction of the ISH signal. Overall the data are consistent with miR-17-92 cluster modulation by Tbx5a also in the cardiac tissue of intact embryos.

These data led us to investigate if the replacement of other cluster members might rescue defects generated by Tbx5a depletion. To address this issue we assayed one member for each of the miRNA families which are present within the miR-17-92 cluster: miR-18a as member of miR-17 family, miR-19b for miR-19 family and miR-92a which belongs to miR-25 family. Data presented in Figure S2 demonstrate that none of the tested miRNA was able to reduce Tbx5a morphant defects.

### miR-19a dysregulation affects zebrafish heart development

**Figure 4:**
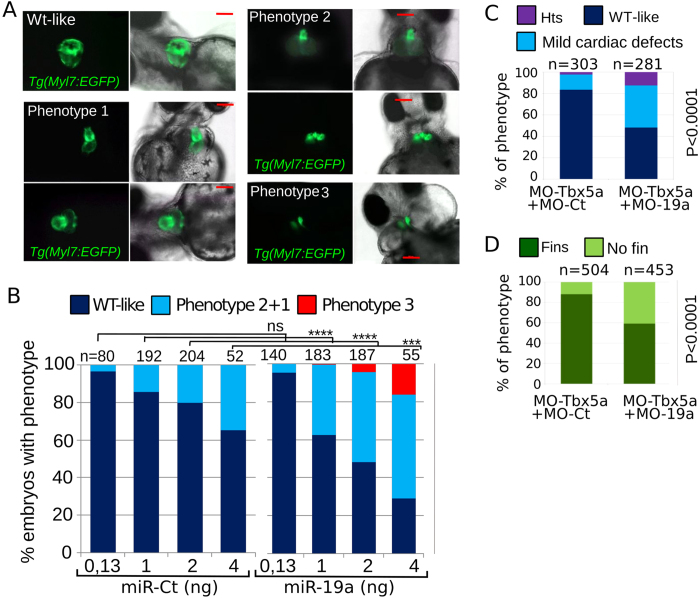
miR-19a dysregulation affects zebrafish heart development. (**A,B**) *Tg(Myl7:EGFP)* embryos were injected with increasing amounts of miR-Ct or miR-19a mimics. (**A**) Images of representative 72 hpf *Tg(Myl7:EGFP)* embryo phenotypes. (**B**) Percentage of embryos with the indicated phenotypes, averaged across multiple independent experiments carried out in double blind. (**D,E**) Phenotypes of 72 hpf *Tg(Myl7:EGFP)* embryos co-injected with 0.5 ng of MO-Tbx5a and with 8ng of MO-19a or MO-Ct. N, total numbers of analyzed embryos. Scale bar 100 μm. hts: heartstring phenotype; mild defects: general cardiac defects; WT: wild type heart. NO fins: pectoral fins absence, WT fins: wild type pectoral fins. P values by Fisher’s test.

In order to investigate the effect of miR-19a dysregulation during early zebrafish development, we performed both gain- and loss-of-function experiments (GOF and LOF). Effects on heart morphogenesis and fin development were assessed by confocal analysis and bright-field microscopy, respectively. Increasing doses of miR-19a injected in *Tg(Myl7:EGFP)* embryos raised the frequency and severity of cardiac defects generated by Tbx5 depletion (Fig. 4A,B). In the less severe phenotype, only looping was affected (phenotype 1, Fig. 4A,B). Increasing the miRNA dose caused shrinkage of the ventricle, atrium bilobation and size reduction (phenotype 2, Fig. 4A,B). In the most severe phenotype, two small deformed hearts were detectable (phenotype 3, Fig. 4A,B). These hearts were still capable of contraction although with a very irregular rhythm. Strikingly, while injection of 2 ng of miR-Ct induced 20% of unlooped hearts, more than 50% of the miR-19a injected embryos presented cardiac defects of variable severity (Fig. 4B). The lowest dose of 0.13 ng, which was able to rescue the Tbx5a morphant phenotype (see Fig. 2A,B) did not cause any apparent cardiac defects (Fig. 4B). Fin lack or defects were never detected in miR-19a overexpressing embryos.

To evaluate the impact of miR-19a dysregulation on vascular integrity we also injected miR-19a mimic in *Tg(flk1:EGFP;myl7:dsRED)* transgenic line. Doses up to 1 ng of miR-19a mimic did not cause visible alterations in vascular structures (Fig. S3).

We performed LOF experiments using a specific morpholino to block the activity of miR-19a (MO-19a),with a non-coding morpholino as control (MO-Ct). Even a high dose (10 ng) of MO-19a did not affect embryo morphology (Fig. S4A,B) but it was able to rescue the morphological defects caused by miR-19a over-expression (Fig. S4C). We speculate that if miR-19a and Tbx5 are part of the same pathway, their LOF effects should be synergic. We injected *Tg(Myl7:EGFP)* embryos with 0.5 ng of MO-Tbx5a. This is a sub-phenotypic dose which generates less than 20% of mild cardiac defects and 10% of embryos without pectoral fins (compare Fig. 4C,D with Fig. 2A,C). Co-injection of the sub-phenotypic dose of MO-Tbx5a with 8 ng of MO-19a strongly increased the number of heart and pectoral fin defects suggesting a synergistic interaction between miR-19a and Tbx5a. Although miR-19a down-regulation alone was not sufficient to trigger the HOS phenotype in zebrafish, these data indicate that dysregulation of miR-19a level impacts heart development.

### Genome-wide analysis of miR-19-induced rescue of Tbx5 morphants

**Figure 5:**
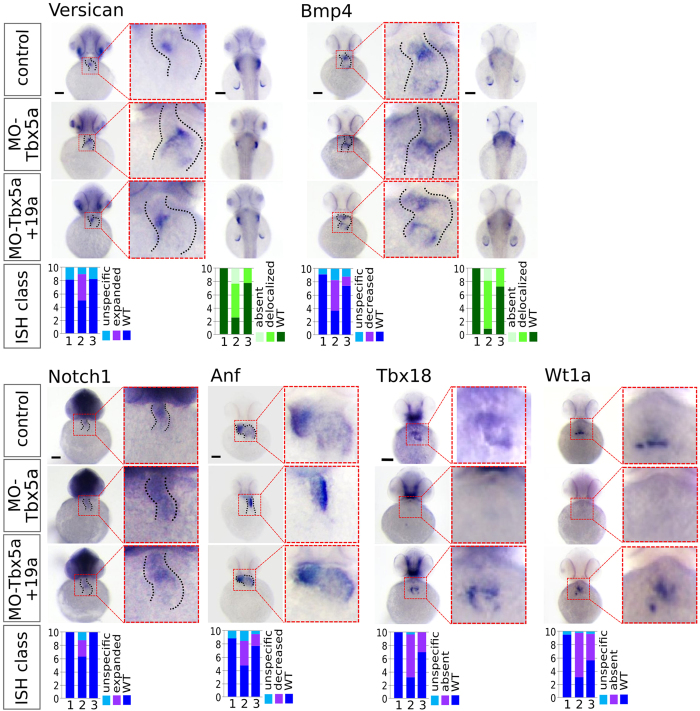
miR-19a replacement partially rescues cardiac marker expression in Tbx5 morphants. WT embryos were co-injected with 1.5 ng of MO-Tbx5a and 0.25 ng of miR-Ct (MO-Tbx5a) or 0.25 ng of miR-19a mimics (MO-Tbx5a + miR-19a). WT embryos co-injected with 1.5 ng of MO-Ct and 0.25 ng of miR-CT were used as control. At 48 hpf, 15–20 embryos from each thesis were fixed for ISH analysis. A small number of embryos were also grown up to 72 hpf and screened by optical microscopy to verify that the percentages of embryos with cardiac defects and fin absence were the expected. Whole mount ISH. Ventral view of 48hpf embryos is shown. For versicana and bmp4 probes, dorsal view is also shown (right side) to highlight the fin bud (only bmp4) and fin apical fold signals. Black scale bars:100 μm. At bottom of the figure, quantifications of different hybridization signals were reported. 1 = MO-Ct + miR-Ct; 2 = MO-Tbx5 + miR-Ct and 3 = MO-Tbx5 + miR-19a.

To characterize the impact of miR-19a on molecular HOS phenotype, we focused on cardiac markers known to be deregulated in Tbx5a morphants/hts mutants. The Tbx5 target natriuretic peptide precursor A (Anf)^29^ and the sinus venosus (SV)- atrioventricular junction (AVJ) differentiation marker bmp4^30^ have been previously shown to decrease their expression after Tbx5a depletion^20^,^23^. On the other hand, notch1b and versican a (Vcana) showed expanded expression compared to the WT in which the expression is restricted to the AVJ^18^,^20^,^30^. Vcana and bmp4 were also exploited as pectoral fin development markers because of their expression in the apical district and within the fin buds in case of bmp4^30^,^31^. A high percentage of Tbx5a morphants co-injected with miR-19a mimic showed at 48hpf a recovered expression of the tested genes in both, cardiac and fin districts (Fig. 5). In addition, the recovery of the WT expression of the proepicardial markers Tbx18 and Wt1a indicates that the replacement of miR-19a is able to rescue the absence of proepicardial specification which is a feature of hts mutants^32^ (Fig. 5). We also analyzed the effect of miR-19a co-injection on valve morphology using the hemizygous *Tg(tie-2:GFP)* line, which allows to visualize the valve tissue. While alteration of Tbx5 expression causes an expansion of Tie2 expression in zebrafish embryos^18^, miR-19a replacement was able to restore the Tie2 expression in a high percentage of Tbx5a morphant*s* (Fig. S5).

**Figure 6:**
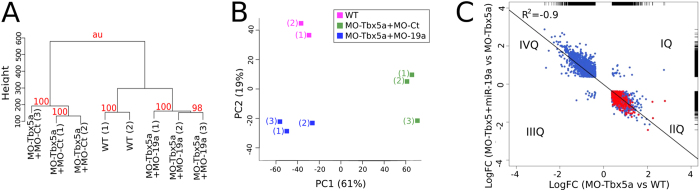
Genome-wide analysis of miR-19-induced rescue of Tbx5 morphants. At 24 hpf about 50 embryos from MO-Tbx5a embryos, MO-Tbx5a + miR-19a embryos and control embryos (generated as described in legend of Fig. 5) were collected and total RNA was extracted for microarray analysis. (**A,B**) The dendrogram from the hierarchical clustering analysis (**A**) and first two PCs from PCA analysis (**B**) are shown. Scores on each dendrogram node in A report the approximately unbiased (AU) probability values as implemented in CONSEL^56^ and represent the statistical significance on a 1 to 100 scale estimated by bootstrap analysis. (**C**) Log2 (FC) of differentially expressed transcripts are shown: MO-Tbx5a/miR-Ct (x axis) and MO-Tbx5a + miR-19a/MO-Tbx5a (y axis). Thick marks along the sides are rug plots which explain the density of the represented data. Selected putative miR-19 targets are shown in red. R^2^ is the square of the Pearson correlation coefficient.

Furthermore, we explored the impact of miR-19a replacement at the global gene expression level, by comparing the microarray profiles of 24hpf zebrafish embryos co-injected with MO-Ct and miR-Ct (WT phenotype) to the profiles of MO-Tbx5a morphants co-injected with miR-Ct (hts phenotype), or with miR-19a (rescued phenotype). We first investigated the overall relationship between sample expression profiles using unsupervised hierarchical clustering analysis based on probe intensity signals. The dendrogram in Fig. 6A shows that, at probe-level, the genome-wide expression profile of rescued embryos is more similar to embryos with WT phenotype rather than to embryos presenting the hts phenotype. The reliability of the obtained cluster was confirmed by bootstrap analysis^33^ (see Fig. 6 legend and Supplementary Methods for details). In addition we employed principal component analysis (PCA) of probes log-transformed expression values to elucidate the main sources of variation. In the space of the first two principal components (PCs), which account for 61% (PC1) and 19% (PC2) of the total variance, the biological replicates of the three conditions were well-separated (Fig. 6B). PC1 discriminates *hts* from WT while rescued embryos are close to the WT on this axis (Fig. 6B). PC2 instead discriminates WT from morpholino-injected embryos independently from the miR-19a status.

Next, we measured the impact of Tbx5a depletion on the global gene expression patterns of embryos treated and untreated with miR-19a. We first identified genes that were differentially expressed (expression FC > 1.3, p < 0.05, FDR < 0.05) in MO-Tbx5a morphants compared to the MO-Ct. Then we intersected the identified genes with the set of differentially-expressed genes in embryos with rescued profiles compared to MO-Tbx5a morphants. We found 4963 genes, corresponding to 59,6% of the gene set altered in MO-Tbx5a embryos, that had significant changes in their expression in rescued embryos. Those genes and their log2 FC are shown in Fig. 6C. Interestingly, most of them (98,7%) resulted inversely modulated (see IIQ and IVQ in Fig. 6C) and their relative expression levels were highly anticorrelated (Pearson R^2^ = −0.9, p < 2.2e^−16^). Since those genes are both direct and indirect targets of miR-19a, in order to identify putative direct targets, we focused on transcripts down-regulated in miR-19a-coinjected Tbx5 morphants vs Tbx5 morphants (IIQ). Among the targets predicted by two algorithms (TargetScanFish and Pita) we found 293 transcripts (shown in red in Fig. 6C, Table S1) which were not only down-regulated in MO-Tbx5 + miR-19a embryos compared to MO-Tbx5 morphants, but also up-regulated in Tbx5 morphants vs control embryos. These transcripts represented an enriched subset (hypergeometric test p = 0.0001) compared to the background of predicted miR-19a targets for all expressed transcripts measured using the array. On the other hand, the number of predicted putative miR-19a targets in the IVQ (transcripts downregulated in MO-Tbx5 morphants and upregulated by the presence of miR-19a) is below the expected background frequency (hypergeometric test p < 2.2e^−16^). Furthermore, to evaluate the influence of modulated miR-19a targets on discriminating between *hts* and WT phenotypes, we selected from PCA the probes with the relative highest absolute loadings (10^th^ percentile) along the PC1. We then selected 7834 probes, accounting for 49.1% of the total weight. These probes corresponded to 5552 unique genes and all the 293 predicted mir-19a targets were included in this list. The most significantly represented biological processes (p < 0.001) in this gene set, based on Gene Ontology (GO) classification^34^ were developmental process, multicellular organism and anatomical structure development (Fig. S6).

Q-RT-PCR analysis performed on 7 selected genes validated the array results (Fig. S7A). Among them the cardiac regulators MEF2Ca and Camk2n1a were tested by means of Dual Luciferase assays carried out on cloned 3’UTR. The ability of miR-19a to reduce the luciferase activity in a dose-dependent manner support its direct control on these crucial cardiac genes (Fig. S7B).

## Discussion

In summary, our data indicate that miR-19a acts as a Tbx5 negative effector regulating several aspects of cardiovascular development.

Despite substantial progress in the comprehension of main roles played by transcription factors in cardiac development, there are gaps in our understanding of the interconnections between cardiogenic transcription factors and their downstream effector genes. Tbx5 is a crucial player in heart development. A network of cardiac transcription factors has been suggested as responsible of the complex Tbx5-dependent regulation^17^. In this work, using zebrafish as HOS model system, we show that miR-19a has a crucial role in the Tbx5 regulatory network. We found that miR-19a is down-regulated in Tbx5 depleted zebrafish embryos and that Tbx5a modulation affects the expression of the miR-17-92 cluster, which hosts miR-19a. Importantly, we showed that modulating miR-19a levels partially rescues cardiac and pectoral fin defects due to Tbx5 downregulation and increases the viability of Tbx5 morphants.

The miR-17-92 cluster has been largely studied as oncogene in tumor and, more recently, its role in cardiovascular system has been also highlighted^35^,^36^. This cluster plays a role in angiogenesis^37^ and it is able to induce cardiomyocyte proliferation in post-natal and adult hearts^38^. While Ventura in 2008 showed that deletion of miR-17-92 in mice causes ventricular septal defects, Danielson *et al*., in 2013 observed that overexpression of this cluster in cardiac and smooth muscle tissues induces dilated, hypertrophic cardiomyopathy, and arrhythmia^27^,^39^. These data highlight the importance of this cluster in regulating cardiac development. Moreover, very recently, Han *et al*.^40^ reported also forelimb defects in miR-17 ~ 92 deleted mice. More specifically, limb and digit malformations are some of the consequences of monoallelic microdeletions involving the miR-17 ~ 92 cluster in a subset of individuals with Feingold syndrome^40^,^41^.

Our data show that, although Tbx5 is able to modulate the miR-17-92 cluster, only miR-19a is effective in rescuing cardiac and fin defects of Tbx5a morphants. Also mir-19a is the only member of the miR-17-92 cluster which resulted significantly decreased in the NGS analysis by Tbx5 depletion. This difference among miRNAs of the same cluster might be due to the extensive post-transcriptional regulation which characterizes miRNA maturation^42^,^43^.

In accordance with a cardiac role of the miR-17-92 cluster, we showed that dysregulation of miR-19a levels alters heart development. Overexpression of miR-19a induces defects of dose-dependent severity ranging from looping defects to cardia bifida (F 4A,B). Recently, overexpression of miR-19b has been shown to impact left-right symmetry and cardiac development of zebrafish embryos; inhibition of the Wnt signaling pathway by miR-19b direct ctnnb1 targeting has been indicated as its possible mechanism^44^. However, in our data the expression of ctnnb1 results unchanged in Tbx5a morphants and it is not decreased by co-injection of miR-19a modulation.

Downregulation of miR-19a by morpholino microinjection was ineffective even using very high doses (10 ng) of MO-19a. In line with this results, Han *et al*., characterizing an allelic series of genetically engineered mice harboring selective targeted deletions of individual components of the miR-17 ~ 92 cluster, showed that the cardiac defects observed in the miR-17~92–null mice^27^ were only detected upon deletion of the entire cluster^40^. A minimal effect of miRNA-mediated loss of function is frequently observed in experimental approaches^45^,^46^,^47^. An explanation for this phenomenon might be the existence of compensatory mechanisms which re-balance a regulatory network when the expression of a single component is slightly altered^47^. However, a general observation is that the miRNA effect can become evident in conditions of stress or injury which causes a strong unbalance^48^,^49^. Our experiments performed with sub-phenotypic doses of MO-Tbx5a (Fig. 4C,D) are in line with these data and support the hypothesis of synergism between miR-19a and Tbx5a in zebrafish.

At genome-wide level, we showed that miR-19a injection is able to rescue the global gene expression profile of Tbx5 morphants while microarray data clearly demonstrate that more than a half of the genes altered by Tbx5a depletion are affected by miR19a. Furthermore, the majority of transcripts significantly upregulated in MO-Tbx5a vs. WT embryos are significantly downregulated in MO-Tbx5a + miR-19a vs. Mo-Tbx5a (Fig. 6C, IIQ) and viceversa (Fig. 6C, IVQ). The remarkable impact that miR-19a has even at the global gene expression level was supported by the observation that putative miR-19a targets are enriched in transcripts upregulated in MO-Tbx5 vs. WT embryos and downregulated by miR-19a co-injection (Fig. 6C, IIQ) compared to the other classes of modulated transcripts.

Among these putative targets MEF2CA, a transcription factor which physically interacts with Tbx5 to synergistically activate gene transcription^50^, has nine putative miR-19 binding sites, making it the best candidate among miR-19a targets. Increasing MEF2C levels in the presence of a Tbx5 decrease might generate a strong unbalance between these two interacting TFs. It has been demonstrated that the relative levels of crucial cardiac TFs working within complexes can be more important for heart development than the absolute levels of these regulators: disruption of these dosage-sensitive interactions could be one of the mechanistic cause of CHDs^51^. Besides MEF2CA, also MEF2AA, which is expressed in zebrafish embryonal cardiomyocytes and it is required for cardiac contractility^52^, is anti-regulated by miR-19. Camk2n1a, an inhibitor of the calcium/calmodulin-dependent protein kinase II(CaMKII), might be also a strong candidate target. CaMKII is necessary for proper heart and fin development and its expression has been shown to be dependent on Tbx5 activity^53^. More specifically, Tbx5 might affect CaMKII expression by acting on its inhibitor through miR-19a modulation. Indeed, dual luciferase assays performed with reporter plasmids carrying the cloned sequences of the MEF2CA-and Camk2n1a-3’UTR support the miR-19a direct control (Fig. S7B). We also identified several potassium and sodium channel forming proteins such as SCN8aa, cacna1ab and kctd8, kctd12.2, kctd15b, this observation is in line with the important role played by Tbx5 in cardiac contractility^54^,^55^. Moreover, the selection of putative miR-19a targets includes also a number of chromatin remodeling proteins (Smarca2, Smarcc1a, Hmga1b, DNMT4, Kdm5ba and Wdr5).

As a whole,these results indicate the complexity of miR-19a functions and its crucial role in the Tbx5 regulatory circuit. Besides providing a tool for identifying new possible targets for HOS, our study in principle shows that modulating miR-19a can positively impact cardiac defects associated with a reduced dosage of Tbx5.

## Materials and Methods

More details can be found in SI Materials and Methods

### ZebrafishLines

Wild-type AB, *Tg(flk1:EGFP), Tg(Myl7:EGFP)* and *Tg(Tie2:GFP)* lines were used in these studies. Zebrafish were raised and maintained under standard laboratory conditions (Westerfileld M zebrafish book) in Zebrafish Housing Systems (Tecniplast, Varese, Italy).

The zebrafish facility, where all the experiments have been performed, is part of the CENTRO di BIOMEDICINA SPERIMENTALE (CBS) of the Area della Ricerca del CNR, Via Moruzzi 1, 56124 Pisa. The zebrafish facility has been authorized by the Italian Ministry of Health with the authorization n°297/2012-A of the 12/21/2012.

The corresponding author declares that all the methods were carried out in accordance with the approved guidelines and that all experimental protocols were approved by the Italian Ministry of Health. Moreover all the technitians and researcher who take care of the animals and perform the experiments are appropriately trained by attending specific courses.

### Microinjection

One-cell stage embryos were injected with a constant injection volume (1 nl, confirmed by volume analysis) using a microinjector (Tritech Research, Los Angeles CA USA). The miRNA mimics used are listed in Table S2.

### Morpholinos

MO-Tbx5a and MO-Tbx5b (Gene Tools) were already described in^18^. The morpholino targeting miR-19a and MO-Ct are the following: MO-miR-19a, CATCAGTTTTG CATAGATTTGCACA; MO-Ct, CCTCTTACCTCAGTTACAATTTATA.

## Additional Information

**How to cite this article**: Chiavacci, E. *et al*. MicroRNA 19a replacement partially rescues fin and cardiac defects in zebrafish model of Holt Oram syndrome. *Sci. Rep*. **5**, 18240; doi: 10.1038/srep18240 (2015).

## Supplementary Material

Supplementary Information
