# Supplementary material for: MicroRNA 19a replacement partially rescues fin and cardiac defects in zebrafish model of Holt Oram syndrome
Source: Sci Rep. 2015 Dec 14;5:18240. doi: 10.1038/srep18240 (PMC4677400; doi:10.1038/srep18240)
Supplement: Supplementary Information [file srep18240-s1.pdf]

Supplementary information

Title:

MicroRNA 19a replacement partially rescues fin and cardiac defects in zebrafish model of Holt Oram syndrome

Authors:

Elena Chiavacci[1♦], Romina D'Aurizio[2], Elena Guzzolino[3], Francesco Russo[2,4], Mario Baumgart[5], Marco Groth[5], Laura Mariani[1], Mara D'Onofrio[6], Ivan Arisi[6], Marco Pellegrini[2], Alessandro Cellerino[7], Federico Cremisi[7], Letizia Pitto[1]

SI Materials and Methods

Reagents

Mature miRNA mimics (dre-miR-19a, dre-miR-19b, dre-miR-219, dre-miR-190, dre-miR-18a, dre-miR-7b, dre-miR-92a and miR-Ct) were synthesized by GenePharma (Shanghai, China); morpholinos (Gene Tools, LLC USA.); Lipofectamine 2000,TRIzol reagent, DNaseI amplification grade, SuperScript II reverse transcriptase, RNAse out, α-minimal Essential Medium (Invitrogen,Life Technologies Italia, Monza, Italy);HyPerFect Transfection Reagent, miRNeasy mini kit, RotorGene, miScript Reverse Transcription kit,Quantitec Reverse Transcription kit and Quantifast SYBR Green PCR kit (QIAGEN, Milan, Italy); anti-DIG antibody-alkaline phosphatase Fab fragment, Blocking reagent, BM Purple and DIG-RNA labeling kit (Roche Diagnostic, Mannheim, Germany); double-DIG miRCURY LNA microRNA 19a detection probe (Exiqon, EUROCLONE, Milano, Italy); SP6 RNA polymerase, RNAse free DNAse I; T7 RNA polymerase (Thermoscientific, Life Technologies Italia, Monza, Italy); Herculase DNA polymerase (Agilent, CA USA); pGEMTeasy vector, Dual Luciferase Reporter System, Glomax luminometer (Promega, Milano Italy); Fetal Bovine Serum (Lonza, Milano Italy); zebrafish diet (SDS, Dietex, France); Tetramisole (Sigma-Aldrich, USA); mMESSAGEmMACHINE® Kit (Applied Biosystems,,Life Technologies Italia, Monza, Italy).

Optical Microscopy and Confocal Analysis. Optical microscopy was performed with Leica M80 microscope, images were acquired with Nikon DS-Fi1 camera and NIS-Elements F 3.0 software. Fluorescence microscopy was performed with Leica DM IL microscope and with Nikon YFL microscope both equipped with CoolSnap CF camera (Photometric). Images were processed with Gimp-2.6 software. For confocal analysis embryos were fixed in 4% PFA for 1 h at room temperature under slow agitation and embedded in 1% low-melt agarose. Images were acquired with a Leica DM IRE 2 confocal microscope. Image stacks were processed with FIJI-WIN32 by projection.

Whole Mount In Situ Hybridization. Whole mount In Situ Hybridization (ISH) was performed as previously described [1]. Whole mount ISH for miRNA was performed as described in [2] with some modifications: for the fixation step the N-(3-Dimethylaminopropyl)-N-ethylcarbodiimide hydrochloride (EDAC) was used. As miR-19a probe a double-DIG miRCURY LNA Detection probe (Exiqon) was used. Hybridization temperature was 37°C.

Cell Culture and Transfection. P19CL6 and HL1 were obtained respectively from Dr. Baldini (Telethon Institute of Genetics and Medicine**,** Napoli, Italy) and from Prof. Claycomb (Department of Biochemistry and Molecular Biology, Louisiana State University Medical Center, LA 70112 USA). Cells were cultured as previously described[3-5]. The experiments for Tbx5 up or down regulation were performed as described [1].

Dre-miR-17-92 promoter cloning and HL1 transactivation experiments. The genomic sequence upstream the miR-17-92 cluster was scanned for Tbx5 consensus binding sites using the TRANSFAC program [6]. A 1.6 kb genomic fragment upstream of dre-miR-17-92 cluster spanning -4300 to -2700 was amplified**,** cloned into pGEM-T Easy vector (Promega) and successively subcloned in pGL3-promoter vector (Promega) to generate the pGL3-MIR vector. HL1 cells were transfected with 500 ng of pGL3-MIR, 100 ng of pRL-TK vector and increasing doses of pcDNA-Tbx5 or empty pCMV vectors using Lipofectamine (LifeTechnologies). Luciferase assay was performed as already described [7].

MEF2Ca and Camk2n1a 3'UTR cloning and dual-luciferase reporter assay. A ~1.5 kbp of MEF2Ca 3′UTR and ~1-kbp of Camk2n1a 3'UTR were amplified from zebrafish genomic DNA and cloned into the Kpn I and Xho I sites of pGLU Dual-luciferase reporter plasmid[8]. HL1 cells were transfected with 100 ng of MEF2Ca-3'UTR-GLU or 100 ng of Camk2n1a-3'UTR-GLU, 100 ng of pRL-TK vector and increasing doses of miR-19a mimic using Lipofectamine (Life Technologies). Luciferase assay was performed as already described [7].

RNA extraction, Quantitative Real Time RT-PCR. Total RNA was extracted using the miRNeasy Mini kit (Qiagen) and quantified using a NanoDrop-1000 spectrophotometer and quality was monitored with the Agilent 2100 Bioanalyzer (Agilent Technologies, Santa Clara, CA). cDNA was retro-transcribed using miScript Reverse Transcription kit (for miRNA analysis) and Quantitec Reverse Transcription kit (for gene analysis). Real-time PCR (qRT-PCR) was carried out using QuantiFast SYBR Green kit with Rotor gene (Quiagen). Primers sequences are listed in table S1. Relative quantification of gene expression was calculated as described[9].

cDNA library preparation and sequencing. For small RNA sequencing, small RNA cDNA libraries were prepared as follows: for each library 1 μg of total RNA was submitted to the small RNA v1.5 sample preparation protocol (llumina Inc., San Diego, USA). In brief, the library preparation was performed as follows: RNA was ligated with adapters to the 5' and 3' termini of the RNA. The adapter ligated samples were used as templates for cDNA synthesis. The cDNA was amplified with 13 PCR cycles to produce sequencing libraries. cDNAs with size between 90 to 100

nt were purified independently for each sample by 10% Novex TBE polyacrylamide gel electrophoresis (Invitrogen) and eluted into 300 µl elution buffer (Illumina) for at least 2 hours at room temperature, to enrich for molecules containing inserts in the range of 18–33 nt. The resulting gel slurry was passed through a Spin-X filter (Illumina) and precipitated by the addition of 20 µg glycogen, 30 µl of 3M NaOAc, pH5.2, and 975 µl of pre-chilled (-20°C) ethanol. After washing with 70% ethanol, the pellet was dried at 37°C for 5-10 min and dissolved in 10 µl resuspension buffer (Illumina). The purified libraries were quantified on the Agilent DNA 1000 chip, diluted to 10 nM and subjected to sequencing-by-synthesis on IlluminaGaIIx.

Analysis of sequencing data. Individual sequence reads with base quality scores were produced by Illumina sequencing. The data was analyzed by the use of CLC-worbench (CLCbio, Arhus, Denmark). After eliminating reads with low quality and trimming the 3' adaptor sequence, the remaining 18- to 33-nt reads were grouped into unique sequence clusters. To exclude potential sequencing errors, only sequences with at least 30 occurrences were included. Annotation of sequence clusters was performed, allowing 2 mismatches and up to 2 additional bases at each end, by using the *Danio rerio* reference from miRBase v16.0. Expression values were normalized to reads per million (rpm).

Microarray. Cyanine-3 (Cy3) labeled cRNA was prepared from 200 ng RNA using Low Input QuickAmp Labeling Kit One-Color (Agilent Technologies) according to the manufacturer's instructions, followed by RNAeasy column purification. Microarray hybridizations were carried out in Agilent's Sure Hyb Hybridization Chambers containing 1650 ng of Cyanine 3-labelled cRNA per hybridization. The hybridization reactions were performed at 65°C for 17 hours using Agilent's Gene Expression Hybridization Kit with the 4X44K whole Danio rerio genome chip (grid ID 026437, G2519F chip). After hybridization, slides were washed with Agilent Gene Expression Wash Buffer 1, Gene Expression Wash Buffer 2 and Acetonitrile for one minute at room temperature. Chips were scanned on an Agilent G2505C scanner. Limmapackage [10] from R/Bioconductor was used to analyse microarray experiments.

Microarray analysis. Limmapackage [10] from R/Bioconductor was used to analyse microarray experiments. The background raw median signals were first adaptively corrected (normexp method and offset=16) [11]. Normalization between arrays was performed using cyclic loess [12] for 35073 probes over 39162 corresponding to 21956 unique gene IDs. The averaged intensities were fitted to multiple linear regression models and an empirical Bayes method was used to estimate moderated t-

statistics, as implemented by Limma. P-values were inferred from moderated t-statistics and the Benjamini and Hochberg's method was used to control the false discovery rate.

Hierarchical clustering was performed on the intensity values of all probes in the array after background correction and cyclic loess normalization. The Euclidean distance was used as metric and Ward as linkage method. The R module pvclust [13] was used to assess statistical significance with multiscale bootstrap resampling of 20,000 iterations [14] PCA analysis was performed using log2-transformed probes intensity signals.

The full small RNAseq and microarray data from the study is available from the GEO database (www.ncbi.nlm.nih.gov/geo, accession number GSE69691######).

Targets prediction. The 3'UTR of genes that resulted down-regulated in miR-19a-coinjected Tbx5 morphants and up-regulated in Tbx5 morphants (Fig.6C,IIQ) were retrieved from Ensembl Biomart and analyzed using TargetScan Fish 6.2 [15] and Pita algorithms [16] to identify putative miR-19a targets. The Ensembl transcript Ids were obtained using g:Convert, a gene id converter tool (http://biit.cs.ut.ee/gprofiler/gconvert.cgi).

Statistical Analysis. Data were analyzed using GraphPad Prism (GraphPad Software, San Diego, CA USA). Statistical differences were determined by unpaired *t*-test, and Fisher's test with values of $P<0.05$ considered statistically significant. Log-rank (Mantel-Cox) Test analysis was used to compare viability curves in figure 2D. Each experimental point in the graph represents the mean ± SE of at least three independent experiments**.**

Table 1 List of putative miR-19 targets identified among the transcripts upregulated in MO-Tbx5a vs MO-Tbx5-Ct and downregulated in MO-Tbx5a+miR-19a vs MO-Tbx5a+miR-Ct.

**Primary Acc**	**Gene Symbol**	**logFC MO-Tbx5a+miR-19a *vs* MO-Tbx5a+miR-Ct**	**logFC MO-Tbx5a *vs* MO-Tbx5-Ct**
BC077081	nrarpa	-1,182728308	1,231138611
BC097003	srsf5b	-1,059491134	0,780031963
BC125839	shoc2	-1,389545757	0,769762422
BC154174	ube2kb	-0,627003303	0,415424597
BC165474	camk2n1a	-1,139668816	0,618350816
DQ402073	zbtb4	-1,513786414	1,138556571
ENSDART00000006221	nbeal1	-0,665237612	0,689541808
ENSDART00000010846	LOC101886726	-0,572447285	0,431134997
ENSDART00000014190	sec31b	-0,97245735	1,005456962
ENSDART00000014465	hip1	-0,856515818	1,0550721
ENSDART00000016230	plxna4	-0,84124452	0,617244139
ENSDART00000017726	etnk1	-1,357275572	0,729812318
ENSDART00000019481	itpkb	-0,440868811	0,711802458
ENSDART00000024193	LOC557995	-0,648890796	0,763016153
ENSDART00000024331	glsb	-1,31015421	0,972324198
ENSDART00000027459	grk6	-0,506774873	0,451547589
ENSDART00000038401	si:ch211-95j8.2	-0,407433628	0,707974627
ENSDART00000038430	akap13	-1,119310725	1,124348208
ENSDART00000040534	rgs12a	-0,673709047	0,510559948
ENSDART00000040707	si:dkeyp-118b1.2	-0,893248257	0,63032988
ENSDART00000041035	snap91	-1,541648403	1,304385424
ENSDART00000047208	madd	-1,118648166	0,890442928
ENSDART00000054092	LOC100536827	-1,077816621	1,204433454
ENSDART00000055176	epn2	-1,461326979	1,367895427
ENSDART00000055336	dennd1a	-0,379652332	0,398564662
ENSDART00000057268	greb1l	-0,479085457	0,660291652
ENSDART00000061738	im:7143453	-0,771221954	0,776158151
ENSDART00000062525	acbd3	-0,893722563	0,600027351
ENSDART00000062631	hapln1b	-0,576855061	0,497326532
ENSDART00000066891	gsap	-0,726800827	0,939329682
ENSDART00000067698	hoxc6b	-0,978983758	0,757829213
ENSDART00000075978	LOC560411	-1,726348075	1,282749316
ENSDART00000078325	LOC557884	-0,934372238	2,229935081
ENSDART00000079080	ppargc1b	-0,6771192	0,553168229
ENSDART00000079131	cdyl	-1,007382655	0,963267902
ENSDART00000079383	hoxa9b	-0,92023392	0,653909595
ENSDART00000080679	LOC100330186	-0,73944585	0,664684796
ENSDART00000080781	arl15b	-0,573178637	0,40490882

ENSDART00000082830	LOC562097	-0,576292353	1,180984934
ENSDART00000083898	ppap2b	-1,004743204	0,630795847
ENSDART00000084501	mpp5a	-0,59404548	0,580612791
ENSDART00000084932	map3k3	-0,477880379	0,548782476
ENSDART00000085996	fermt2	-0,524835975	0,520960649
ENSDART00000086206	atxn1b	-0,982255935	1,025791507
ENSDART00000086734	phldb1a	-0,877523102	0,658083584
ENSDART00000087204	dusp3a	-0,78384948	0,464081495
ENSDART00000087610	arhgef12	-0,6825531	0,543973593
ENSDART00000088808	usp32	-0,927489934	1,01863875
ENSDART00000091105	LOC557798	-0,664657677	0,470838817
ENSDART00000091612	dab2ipa	-0,781950063	0,696808732
ENSDART00000097649	clpxb	-0,608121101	0,577181529
ENSDART00000100214	nufip2	-1,132089114	1,162806636
ENSDART00000100798	LOC792697	-0,450015757	0,518454971
ENSDART00000101463	zgc:136739	-0,812109116	1,023217637
ENSDART00000101707	dhx40	-0,583359576	0,484995205
ENSDART00000102969	spock3	-1,586639394	1,014265429
ENSDART00000104299	LOC101884361	-0,870912425	1,214838007
ENSDART00000104799	col11a2	-1,241737336	1,178256933
ENSDART00000105533	enthd2	-0,49987988	0,417606386
ENSDART00000105691	LOC402880	-1,50976206	1,13259104
ENSDART00000109165	cpz	-1,023426809	0,740982182
ENSDART00000109268	LOC100003958	-0,633013403	0,588250798
ENSDART00000109333	LOC100334507	-0,60346207	0,416711257
ENSDART00000109576	faah	-0,566490634	0,646598302
ENSDART00000109911	hgsnat	-0,729030982	0,816986111
ENSDART00000110907	itfg1	-0,673167045	0,452599566
ENSDART00000111080	bai3	-0,806136025	0,637292433
ENSDART00000111438	mgea5	-0,94749936	0,717982074
ENSDART00000111837	raver2	-0,557611835	0,405708616
ENSDART00000111894	si:ch73-138n13.1	-0,877966846	0,492845155
ENSDART00000111956	gramd1a	-0,491503571	0,44692623
ENSDART00000112414	rapgef5a	-2,417870603	2,022607158
ENSDART00000112694	LOC797104	-1,600331812	1,038336455
ENSDART00000112873	LOC567642	-0,400989828	0,559062753
ENSDART00000113294	fzd3a	-1,552734832	1,22508213
ENSDART00000113461	pcdh11	-1,4017623	0,874666289
ENSDART00000113893	tmco4	-0,922759245	0,643753435
ENSDART00000114190	ppp1r9ba	-0,572656079	0,418260181
ENSDART00000114222	LOC100334650	-0,440366442	0,435373214
ENSDART00000114561	LOC100536336	-0,890110954	0,756595876
ENSDART00000122519	st8sia6	-0,426908803	0,721557013
ENSDART00000122796	rgl3a	-0,553763607	0,406285996
ENSDART00000123469	clcn3	-0,476731078	0,630418469

ENSDART00000123809	LOC796144	-1,085650705	0,95448797
ENSDART00000124505	bahd1	-1,179037043	0,812821855
ENSDART00000125043	smad1	-0,839188869	0,978312928
ENSDART00000125425	snap47	-1,040229933	0,987009291
ENSDART00000125804	rbms3	-0,477796487	0,502497266
ENSDART00000125824	tmem198a	-0,829473068	0,561687085
ENSDART00000126317	cab39	-0,603122121	0,726413139
ENSDART00000126440	LOC793308	-0,480881236	0,422571649
ENSDART00000126480	LOC100330779	-1,795686303	2,253490675
ENSDART00000127167	fam217b	-1,071526776	0,502182744
ENSDART00000128415	LOC100537923	-0,699753641	0,380057535
ENSDART00000128903	LOC100534965	-0,446003784	0,73240495
ENSDART00000128947	kmt2bb	-0,9885809	0,728113624
ENSDART00000129134	pard3	-1,039873162	1,035724592
ENSDART00000129834	LOC100003245	-0,810026632	0,507843836
ENSDART00000130278	LOC561649	-0,79944154	0,752035861
ENSDART00000131128	LOC100006622	-0,572639445	0,500313687
ENSDART00000131310	si:dkey-93n13.2	-0,429134642	0,464267902
ENSDART00000133851	LOC100535812	-0,472578604	0,432213317
ENSDART00000136146	nf2b	-1,205144128	0,905030446
ENSDART00000137373	si:ch211-266i6.3	-1,0642675	0,663320413
ENSDART00000138128	si:ch73-112l6.1	-0,57160403	0,431459746
ENSDART00000140301	LOC560706	-0,954988026	1,056824676
ENSDART00000141203	sh3bp5lb	-0,643167476	0,473323314
ENSDART00000141338	LOC559101	-0,525348518	0,818438088
ENSDART00000146052	man1a1	-0,625114696	0,44971932
ENSDART00000146180	csrnp2	-0,90131648	0,689480742
ENSDART00000149903	LOC562945	-0,839895826	0,570778433
ENSDART00000150143	tead1b	-1,099745664	0,416153634
ENSDART00000150958	lrrc4ba	-0,855537915	0,47209425
ENSDART00000151938	si:ch211-236l14.3	-0,457292162	0,527503059
ENSDART00000152019	si:ch211-234p6.5	-0,798916954	0,572478009
ENSDART00000152028	LOC569952	-0,965994049	1,32522273
ENSDART00000152428	LOC562449	-0,692214158	0,398818997
ENSDART00000152991	nfe2l1b	-0,720136909	1,03459186
ENSDART00000154053	si:ch73-147o17.1	-0,571100397	0,494277263
ENSDART00000154978	LOC101884856	-1,03093447	0,535135323
ENSDART00000155549	si:dkey-6e2.3	-1,137406873	1,017338431
ENSDART00000156078	im:6907928	-1,242371118	0,713057964
ENSDART00000156705	si:dkeyp-72g9.4	-0,86746619	0,417388128
NM_001001815	igf2b	-0,476176434	0,444431697
NM_001001822	ptenb	-0,636010574	0,475456181
NM_001002055	cops2	-0,513100237	0,385142938
NM_001002103	atg4c	-1,204175769	1,00561453

NM_001002406	nr4a2b	-0,92651956	0,491418961
NM_001002656	dgcr2	-0,573032581	0,623537542
NM_001002677	sumo3b	-0,827286506	0,722769019
NM_001003478	prr15la	-1,080348249	1,044664335
NM_001003518	chst1	-0,803376411	0,598109201
NM_001003606	fam49a	-1,508438427	1,026213098
NM_001003624	diaph3	-0,692420391	0,622929296
NM_001003628	crk	-0,878615415	0,755099817
NM_001003870	ppp1r12a	-1,090305565	0,851310224
NM_001003981	ptn	-0,822266655	0,604998799
NM_001004018	gipc1	-0,601439876	0,427146867
NM_001004514	si:busm1-79m10.4	-0,491046817	0,471997302
NM_001004552	pdxdc1	-0,389069925	0,715146681
NM_001004601	nucks1a	-0,597161782	0,66490948
NM_001005948	zgc:103657	-0,846548521	0,457791333
NM_001005966	scocb	-0,797233706	0,791229932
NM_001007030	synj1	-0,622857529	0,424641081
NM_001007359	rab11a	-0,438080493	0,456247065
NM_001007378	pptc7a	-0,652952641	0,677945522
NM_001008624	arhgef7b	-0,800839582	0,549246436
NM_001009914	atg5	-0,52052776	0,776445221
NM_001013300	zgc:112980	-0,398837153	0,404263058
NM_001013310	rtel1	-0,681174051	0,492668137
NM_001013331	zgc:113263	-1,38917424	1,146926979
NM_001013443	eif4g2b	-0,797269119	0,984516178
NM_001014306	lifra	-0,535608717	0,405103394
NM_001015068	sppl3	-0,519883894	0,434064085
NM_001017665	zgc:112052	-0,488762391	0,620844539
NM_001020629	hrasb	-0,596358581	0,511794182
NM_001020679	prelid1b	-1,186805955	0,714181229
NM_001025450	dnmt4	-0,399406459	0,789537886
NM_001025538	tspan3a	-0,486046119	0,568279785
NM_001030246	ddhd1b	-0,734133136	0,553434303
NM_001034019	kctd12.2	-0,847179503	0,630754572
NM_001034976	prkacbb	-0,663576607	0,494866885
NM_001037427	zgc:123060	-0,456165049	0,530936585
NM_001039992	asap3	-0,422218718	0,552596047
NM_001040332	zyg11	-0,43642069	0,60988027
NM_001040341	atxn7l3	-0,822771722	0,872278278
NM_001040393	kalrnb	-1,273248558	0,885486202
NM_001043325	loxl1	-0,381864805	0,887254707
NM_001044311	txlng	-1,010376871	0,717770088
NM_001044799	stim1a	-0,426104124	0,460124485
NM_001044830	zgc:153031	-0,383445266	0,419465336

NM_001044833	dyrk2	-0,817694881	1,079609907
NM_001044865	chka	-0,854831228	0,872509889
NM_001044899	myt1la	-1,055062611	0,456145019
NM_001045056	reps1	-1,020789411	0,830235265
NM_001045094	si:ch211-89p3.3	-0,664886644	1,035930551
NM_001045293	flj11011l	-0,541611112	0,772672103
NM_001045552	mapk6	-1,111061512	0,790689769
NM_001076575	zgc:153725	-0,93633122	0,663169655
NM_001076716	rbms3	-1,069128434	0,853171213
NM_001077147	zgc:153606	-0,737075728	0,410621786
NM_001077212	dctn1a	-0,656842749	0,480705939
NM_001077276	hmga1b	-0,530499186	0,918128388
NM_001077455	ppm1h	-0,449253597	0,380661693
NM_001077560	cluha	-0,507979022	0,439521162
NM_001077603	atf7ip	-0,492292239	0,59329759
NM_001077729	zgc:153952	-0,423853668	0,420945232
NM_001077757	zdhhc3b	-0,889525442	2,799842092
NM_001077766	fam160b2	-0,960257189	0,546380346
NM_001079969	spoplb	-0,762094567	0,818672059
NM_001080029	arrdc3a	-0,608883464	0,504906639
NM_001080177	amotl2a	-0,482010046	0,553580234
NM_001080606	pank2	-0,764664508	0,580459641
NM_001080992	ankib1a	-0,688229652	0,622248819
NM_001082808	pxk	-0,585652072	0,478252135
NM_001083063	capn12	-0,509161198	0,49590522
NM_001083812	ell2	-0,925480586	0,47200112
NM_001083853	pbx1b	-0,422468124	0,449867784
NM_001089336	ptrfb	-0,644338495	1,021794807
NM_001089409	zgc:158364	-0,483631227	0,513157103
NM_001089523	zgc:162267	-0,939051795	0,892638281
NM_001098731	iqca1	-0,805755498	0,458678434
NM_001099246	abca5	-0,945143306	0,631520976
NM_001109724	tead3b	-1,199237153	1,312979306
NM_001110349	vegfaa	-1,614165221	1,59457112
NM_001110458	dis3l	-0,413089953	0,464313045
NM_001110519	atf6	-0,505682162	0,383302655
NM_001113638	zgc:171551	-0,380796794	0,626317295
NM_001115139	clasp1a	-0,710345554	0,55461917
NM_001122613	si:ch211-165d12.4	-0,906810214	0,522370912
NM_001126423	ddx3	-0,491001793	0,474236211
NM_001128242	atp2b3b	-0,574048086	0,41439889
NM_001128248	kcnn1a	-0,691553307	0,485833987
NM_001128327	kdm5ba	-0,892104672	0,61333921
NM_001128337	LOC559976	-0,583368724	0,514395047

NM_001128408	rnf11b	-0,634434075	0,761826665
NM_001128529	mepce	-0,620246611	0,571646524
NM_001128738	fam69b	-0,965402776	0,484359426
NM_001128812	tmem65	-0,985293031	0,720968897
NM_001128825	rab11fip4b	-0,849684658	0,962044712
NM_001136477	mvb12bb	-0,703709649	0,914238835
NM_001142266	pcsk2	-0,613600314	0,441321015
NM_001144053	cdk6	-0,786078165	0,909640815
NM_001159971	spty2d1	-1,012652481	0,592959965
NM_001163291	gpr126	-0,661966168	0,552293539
NM_001166155	npm1b	-0,754695574	0,446176389
NM_001166205	hip1	-0,731762051	0,529051345
NM_001167956	polr2c	-0,846798738	0,61247243
NM_001172629	nr4a3	-0,469302197	0,830534159
NM_001194978	cltcb	-0,577364017	0,388046912
NM_001202440	rnf19b	-0,400902042	0,423025087
NM_001278703	trip11	-0,505772054	0,419866931
NM_001281792	sipa1l1	-0,461907078	0,470488617
NM_130916	inhbaa	-0,57153561	0,473697823
NM_130935	skia	-0,691576311	0,498272351
NM_130943	frzb	-0,502664033	0,526116458
NM_130944	dlc	-0,47553414	0,390814821
NM_131023	efnb2a	-0,571335794	0,508387833
NM_131025	ccnd1	-0,393907159	0,58952883
NM_131044	eng2a	-0,843562434	0,471441037
NM_131237	mxi1	-1,355086147	1,274158611
NM_131275	rxrba	-0,733178515	0,743821918
NM_131312	mef2ca	-0,384499346	0,559746374
NM_131316	ldb1a	-0,40701729	0,44143907
NM_131480	stat1a	-0,888268043	0,528584777
NM_131530	hoxc6b	-1,50561021	1,413980769
NM_131531	hoxc1a	-0,567792288	0,422764352
NM_131584	per3	-0,480477868	0,472849861
NM_131628	scn8aa	-1,124233064	0,953727247
NM_131629	slc40a1	-0,678725629	0,44479749
NM_152977	dmbx1a	-0,534466722	0,693358051
NM_181496	nrarpb	-0,390563958	0,571159032
NM_182889	nfe2l2a	-0,549159918	0,628374765
NM_194402	mknk2b	-1,168784223	1,135079029
NM_194410	vax1	-0,684635505	0,447290195
NM_198068	cxcl12b	-0,683531662	0,513132668
NM_198878	rbpja	-0,568154378	0,388307438
NM_199209	cbfb	-0,491475425	0,454940487
NM_199621	lnpep	-0,416230369	0,443878892
NM_199633	wsb1	-0,752183658	0,802511802

NM_199646	itm2ba	-1,103674935	0,623409258
NM_199682	ap1g1	-0,479284898	0,461121056
NM_199703	tnika	-0,970804486	0,766365444
NM_199705	rbm4.1	-0,760067126	0,59311094
NM_199805	jade1	-0,592459604	0,524393301
NM_199812	zgc:66472	-0,468024897	0,515971443
NM_200155	uso1	-0,435834783	0,507933744
NM_200256	slc25a32a	-0,761614476	0,52921198
NM_200315	zgc:56306	-0,610696383	0,796140994
NM_200385	gorab	-0,536159485	0,489758577
NM_200656	guca1e	-0,739985922	0,983859413
NM_200753	tbcela	-0,841155449	0,485314821
NM_200823	zgc:66125	-0,606397008	0,477033519
NM_201167	hpda	-0,978421627	0,506870294
NM_201209	zgc:64130	-0,445183863	0,693667765
NM_205629	fastkd1	-0,460149658	0,653745956
NM_205728	gli3	-0,865445782	0,856700452
NM_205743	araf	-0,750072324	0,962619309
NM_207052	midn	-1,304113908	1,367777011
NM_212723	mknk2a	-0,398141361	0,527659509
NM_212732	mdm4	-0,553554212	0,401879944
NM_212863	sp7	-0,655789948	0,467325482
NM_212876	cyb5b	-0,391916692	0,505533474
NM_212987	ube2q2	-0,667625167	0,672216643
NM_212997	acbd3	-0,982509643	1,222427679
NM_213001	klhl20	-0,995993533	0,643014083
NM_213048	srfbp1	-0,785116944	0,595660515
NM_213172	ccng2	-0,386495766	0,41898208
NM_213397	pfkfb3	-0,835618073	0,6769655
NM_213408	slc25a22	-0,539524351	0,423712052
NM_213481	gnb1b	-0,633939947	0,627560542
NM_214724	rgs12b	-0,599287643	0,78121173
NM_214756	nnt	-0,601578899	0,42747605

Table S2. Sequence of oligonucleotides used in this work

Gene	***Forward primer***	***Reverse primer***
EF1α	CTGGAGGCCAGCTCAAAC	ATCAAGAAGAGTAGTACCGCTAGCAT
βactin	CGAGCTGTCTTCCCATCC	TCACCAACGTAGCTGTCTTTCTG
actinα2	GCGGTGTTCCCTTCCATA	CGCCTCATCACCAACGTAA
cacnb4a	GGGATAGGCGACGGAGTC	GGCGTGAGTAGGTGCCTCTAT
SRFa	AGCCGTTCCCCTTCATTC	GTGAGGGTGCTACTGCTGCT
Smarca2	AGCCCGTGGTCAGTGATG	GCTTCCTTCTGACTGCTCGT
bmpr1aa	GCGTCAGCTTTTGTTCATC	TGATCAGGATTCTGACCTGCT
MEF2CA	GAAACACAGGAGGTCTGA	GTGGTTTCCGTACCCGTTT
miR-17-92 promoter	*GGTAC*CTTTGCACATTGTC ATTTACATTTG*	TGTTACGAAATTTTACCACATTATTC
MEF2Ca-3'UTR	*GGTACC*TGAACACCATTG ACAAAC*	ACCGTTAGCTGAGGAAACTATG
Camk2n1-3'UTR	*GGTACC*GATTTGAAGCAC TTGTCG*	TCAAGTCACAGTTTACGATTTC

* underlined nucleotides indicate the anchored KpnI restriction site used for sequence cloning or subcloning.

Table S3. Sequence of miRNA mimics used in this work

microRNA	***Sense strand***	***antisense strand***
miR-19a-	UGUGCAAAUCUAUGCAAAACUGAUU	UCAGUUUUGCAUAGAUUUGCUAAUU
miR-7b	UGGAAGACUUGUGAUUUUGUUUU	AACAAAAUCACAAGUCUUAAAUU
mir-130a	CAGUGCAAUGUUAAAAGGGCAUUU	AUGCCCUUUUAACAUUGCAGAGUU
miR-18a	UAAGGUGCAUCUAGUGCAGAUAUU	UAUCUGCACUAGAUGCACCCAAUU
miR-190b	UGAUAUGUUUGAUAUUCGGUUGUU	CAACCGAAUAUCAAACAUAAGAUU
miR-219	UGAUUGUCCAAACGCAAUUCUUU	AGAAUUGCGUUUGGACAAAGAUU
miR-19b	UGUGCAAAUCCAUGCAAAACUGAUU	UCAGUUUUGCAUGGAUUUGCUAAUU
miR-92a	UAUUGCACUUGUCCCGGCCUGUUU	ACAGGCCGGGACAAGUGCAUAAUU
miR-Ct	CUCUAGGUUAAACUCCUGGUU	AACCAGGAGUUUAACCUAAUGUU

Supplementary figure legends

Figure S1.miR-7b, miR-130a, miR-190b and miR-219 are not able to rescue Tbx5a morphant defects. (A-D’) 0.25 ng of the indicated miRNA mimics or miR-Ct were co-injected with 1.5 ng of MO-Tbx5a in *Tg(Myl7:EGFP)* embryos. At 72 hpf embryos were screened by fluorescence microscopy for heart morphology (A-D) and by optical microscopy for pectoral fin presence (A’-D’). Percentage of embryos with the indicated heart defects, averaged across multiple independent coinjections for each miRNA carried out in double blind. hts: heartstring phenotype; mild defects: general cardiac defects; WT: wild type heart. NO fins: pectoral fins absence, WT fins: wild type

pectoral fins. P values by Fisher's test. (E) Global numbers of dead embryos during the first 3 days of development for each microRNA tested.

Figure S2. Members of miR-17-92 cluster, except of miR19a, are not able to rescue Tbx5a morphant defects. 0.25 ng of the indicated miRNA mimics or miR-Ct were co-injected with 1.5 ng of MO-Tbx5a in *Tg(Myl7:EGFP)* embryos. At 72 hpf embryos were screened by fluorescence microscopy for heart morphology (A-C) and by optical microscopy for pectoral fin presence (A'-C'). Percentage of embryos with the indicated heart defects, averaged across multiple independent experiments carried out in double blind (A-C'). Bif: bifid hearts; hts: heartstring phenotype; mild defects: general cardiac defects; WT: wild type heart. NO fins: pectoral fins absence, WT fins: wild type pectoral fins. P values by Fisher's test.

Figure S3. **miR-19a dysregulation does not affect zebrafish vascular development.** Representative images of 72 hpf *Tg(flk1:EGFP;Myl7:dsRED)* embryos injected with 1 ng of miR-Ct (upper) or 1 ng of miR-19a (lower).

Figure S4. **Co-injection of MO-19a is able to rescue cardiac defects induced by miR-19a over-expression**. (A) Confocal images of 72hpf hearts from *Tg(Myl7:EGFP)* embryos injected with 8ng of MO-Ct or 8 ng of MO-19a. (B) Percentage of embryos with the indicated heart defects, averaged across multiple independent experiments carried out in double blind. Unspecific defects: non cardiac defects; No loop: heart with looping defects; WT: wild type heart. (C) Phenotypic analysis of 48 hpf *Tg(Myl7:EGFP)* embryos co-injected with 1ng of miR-19a mimic and 8 ng of MO-Ct or MO-19a. Phenotypes 1, 2+3 are related to images presented in figure 4. P values by Fisher's test.

Figure S5. MiR-19a is able to rescue valve morphology. Confocal images of 72 hpf *Tg(tie-2:GFP)* embryos co-injected with 1.5 ng of MO-Tbx5a and 0.25 ng of miR-19a (C) or miR-Ct (B) mimics. As control, an example of uninjected embryo is also shown (A). In D a quantification of the different phenotypes is reported. Scalebar: 75um. About 20 embryos for each thesis were screened.

Figure S6. Development GO categories are overrepresented among the most influencing genes through first component of PCA analysis. Hypergeometric test was performed to assess over-representation of Biological Process GO categories in selected genes and corrected for multiple test (adjusted p-value < 0.01) through Benjamini & Hochberg [17] for multiple test. Categories with

lowest p-value are shown in green-color scale and the number of genes for each category as bar plot.

Figure S7. miR-19a target validation by quantitative real-time PCR analysis and dual luciferase assay (A) Average changes in gene expression of 7 of the most interesting genes shown to have increased expression in MO-Tbx5 embryos compared MO-Ct embryos and decreased expression in MO-Tbx5+miR-19 compared to MO-Tbx5 embryos by microarray analysis. Values are normalized on EF1 and ß-actin expressions. *P < 0.05. (B) The 3'UTR of MEF2CA and Camk2n1a was cloned in the pGLU Dual-luciferase reporter plasmid and transiently transfected HL1 cells. Increasing amounts of the miR-19a were co-transfected with the luciferase reporter constructs together with the Renilla luciferase pRL–TK vector as internal standard. In each transfection the total amount of the transfected miRNA was kept constant by adding a scrumbled miRNA (miR-Ct) to the specific miRNA to obtain 80 ng. *p<0.05 ; ***p<0.001 compared with luciferase reporter co-transfected with 80 ng of the miR-Ct.

Supplementary references

1 Chiavacci, E. et al. MicroRNA 218 mediates the effects of Tbx5a over-expression on zebrafish heart development. PLoS One 7, e50536, doi:10.1371/journal.pone.0050536 (2012).

2 Lagendijk, A. K., Moulton, J. D. & Bakkers, J. Revealing details: whole mount microRNA in situ hybridization protocol for zebrafish embryos and adult tissues. Biology open 1, 566-569, doi:10.1242/bio.2012810 (2012).

3 Habara-Ohkubo, A. Differentiation of beating cardiac muscle cells from a derivative of P19 embryonal carcinoma cells. Cell Struct Funct 21, 101-110 (1996).

4 Snyder, M., Huang, X. Y. & Zhang, J. J. Stat3 directly controls the expression of Tbx5, Nkx2.5, and GATA4 and is essential for cardiomyocyte differentiation of P19CL6 cells. J Biol Chem 285, 23639-23646 (2010).

5 Claycomb, W. C. et al. HL-1 cells: a cardiac muscle cell line that contracts and retains phenotypic characteristics of the adult cardiomyocyte. Proc Natl Acad Sci U S A 95, 2979-2984 (1998).

6 Pickert, L., Reuter, I., Klawonn, F. & Wingender, E. Transcription regulatory region analysis using signal detection and fuzzy clustering. Bioinformatics 14, 244-251 (1998).

7 Verduci, L. et al. MicroRNA (miRNA)-mediated interaction between leukemia/lymphoma-related factor (LRF) and alternative splicing factor/splicing factor 2 (ASF/SF2) affects mouse embryonic fibroblast senescence and apoptosis. J Biol Chem 285, 39551-39563, doi:10.1074/jbc.M110.114736 (2010).

8 Poliseno, L. et al. Identification of the miR-106b~25 microRNA cluster as a proto-oncogenic PTEN-targeting intron that cooperates with its host gene MCM7 in transformation. Sci Signal 3, ra29, doi:10.1126/scisignal.2000594 (2010).

9 Pfaffl, M. W. A new mathematical model for relative quantification in real-time RT-PCR. Nucleic Acids Res 29, e45 (2001).

10 Smyth, G. K. Linear models and empirical bayes methods for assessing differential expression in microarray experiments. Statistical applications in genetics and molecular biology 3, Article3, doi:10.2202/1544-6115.1027 (2004).
11 Ritchie, M. E. et al. A comparison of background correction methods for two-colour microarrays. Bioinformatics 23, 2700-2707, doi:10.1093/bioinformatics/btm412 (2007).
12 Smyth, G. K. & Speed, T. Normalization of cDNA microarray data. Methods 31, 265-273 (2003).
13 Suzuki, R. & Shimodaira, H. Pvclust: an R package for assessing the uncertainty in hierarchical clustering. Bioinformatics 22, 1540-1542, doi:10.1093/bioinformatics/btl117 (2006).
14 Kerr, M. K. & Churchill, G. A. Bootstrapping cluster analysis: assessing the reliability of conclusions from microarray experiments. Proc Natl Acad Sci U S A 98, 8961-8965, doi:10.1073/pnas.161273698 (2001).
15 Lewis, B. P., Burge, C. B. & Bartel, D. P. Conserved seed pairing, often flanked by adenosines, indicates that thousands of human genes are microRNA targets. Cell 120, 15-20, doi:10.1016/j.cell.2004.12.035 (2005).
16 Kertesz, M., Iovino, N., Unnerstall, U., Gaul, U. & Segal, E. The role of site accessibility in microRNA target recognition. Nat Genet 39, 1278-1284, doi:10.1038/ng2135 (2007).
17 Klipper-Aurbach, Y. et al. Mathematical formulae for the prediction of the residual beta cell function during the first two years of disease in children and adolescents with insulin-dependent diabetes mellitus. Medical hypotheses 45, 486-490 (1995).
18 Klipper-Aurbach, Y. et al. Mathematical formulae for the prediction of the residual beta cell function during the first two years of disease in children and adolescents with insulin-dependent diabetes mellitus. Medical hypotheses 45, 486-490 (1995).

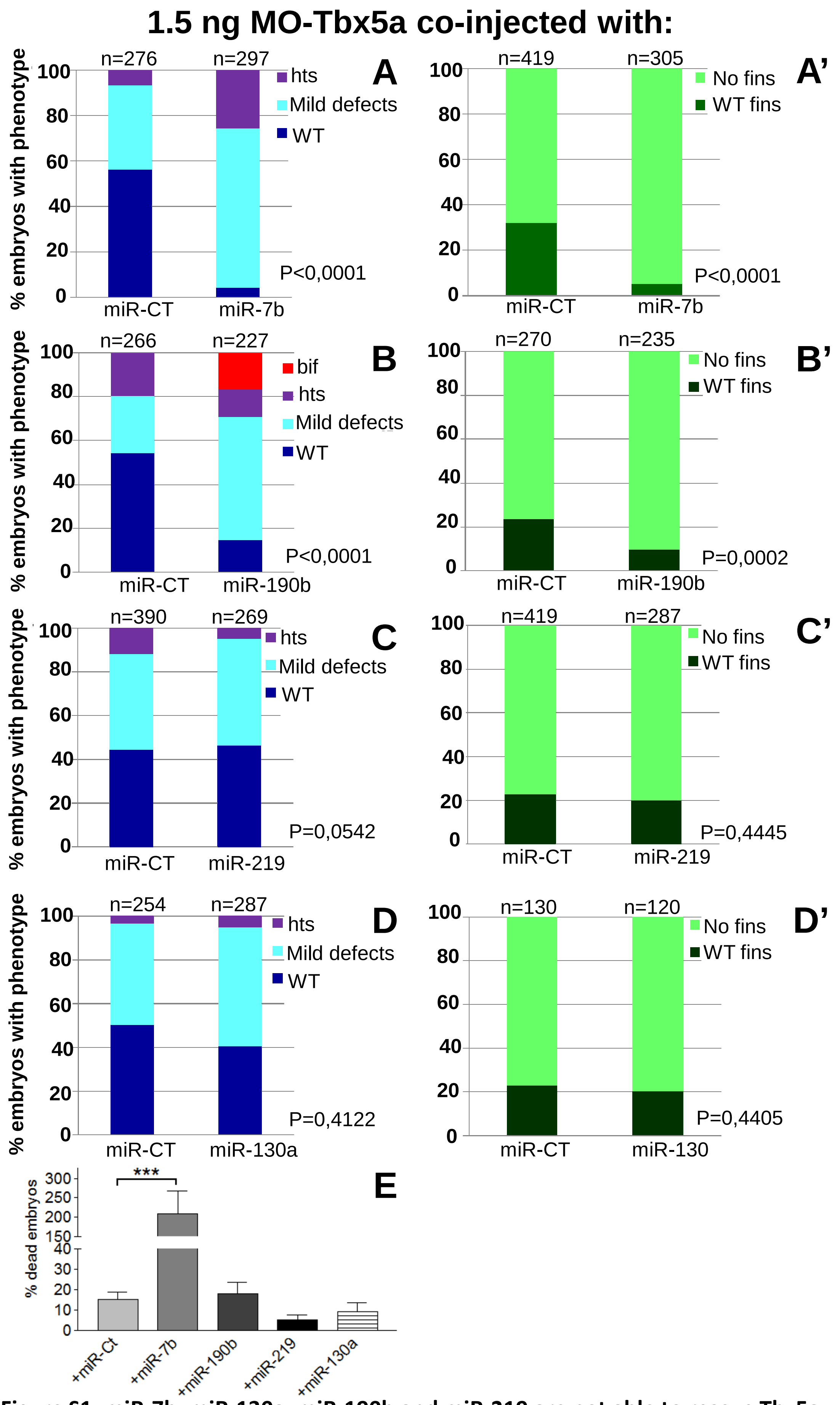

Figure S1. miR-7b, miR-130a, miR-190b and miR-219 are not able to rescue Tbx5a morphant defects.

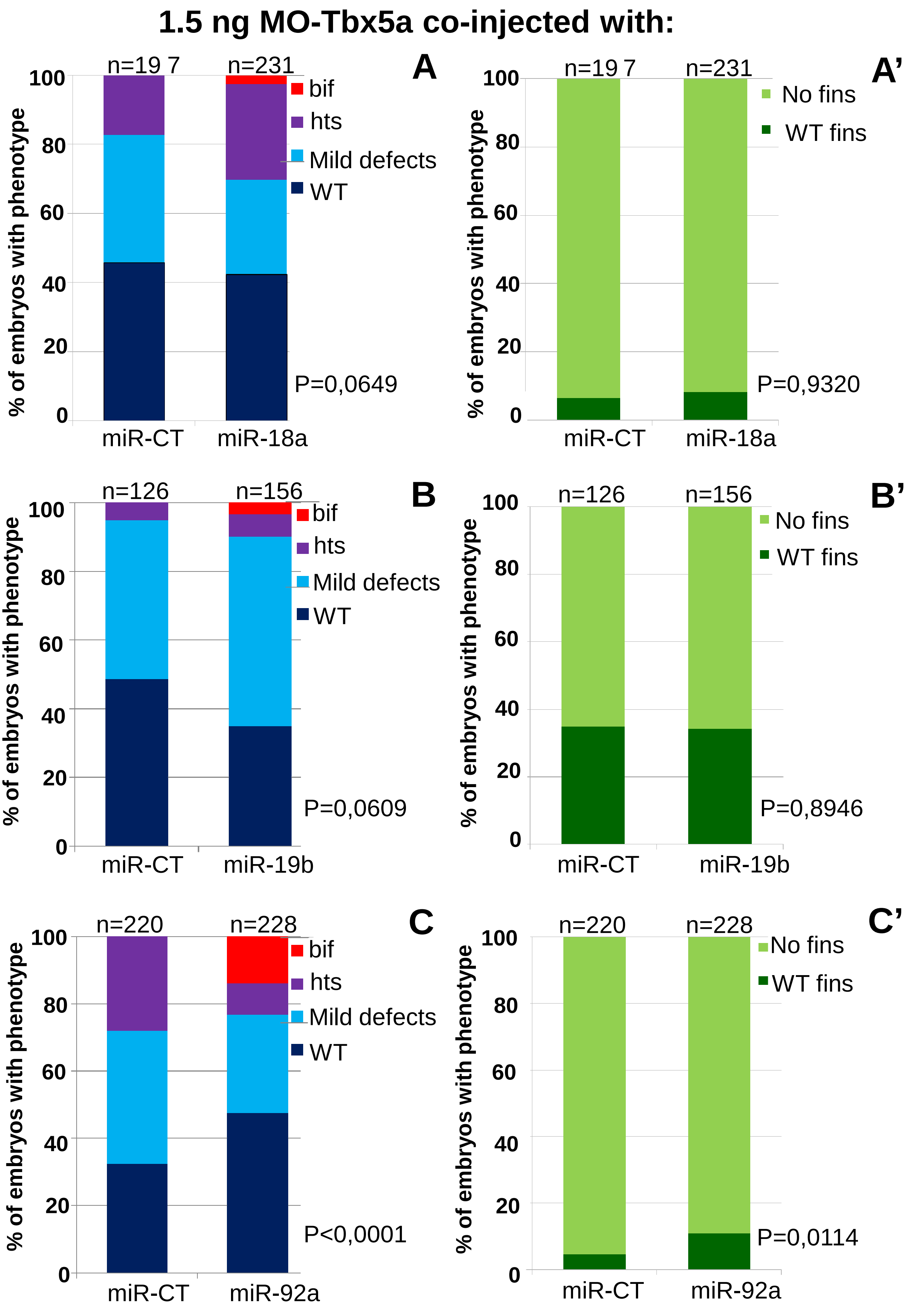

Figure S2. Members of miR-17-92 cluster, except of miR19a, are not able to rescue Tbx5a morphant defects.

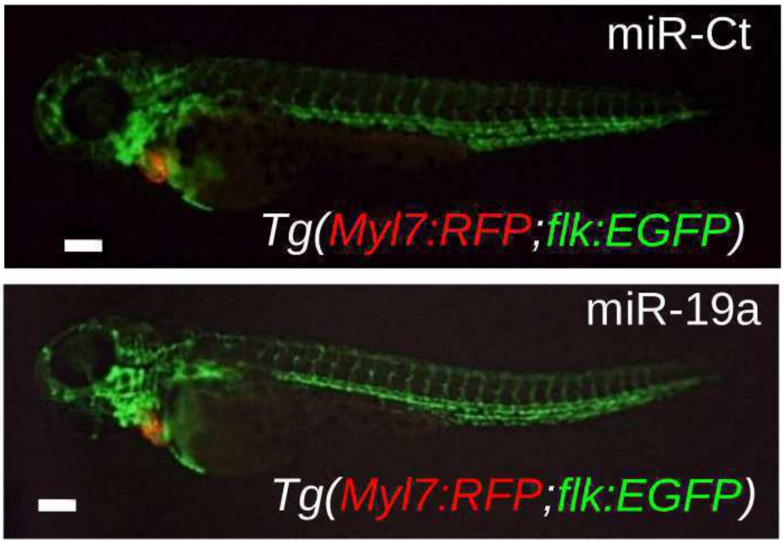

Figure S3. **miR-19a dysregulation does not affect zebrafish vascular development.**

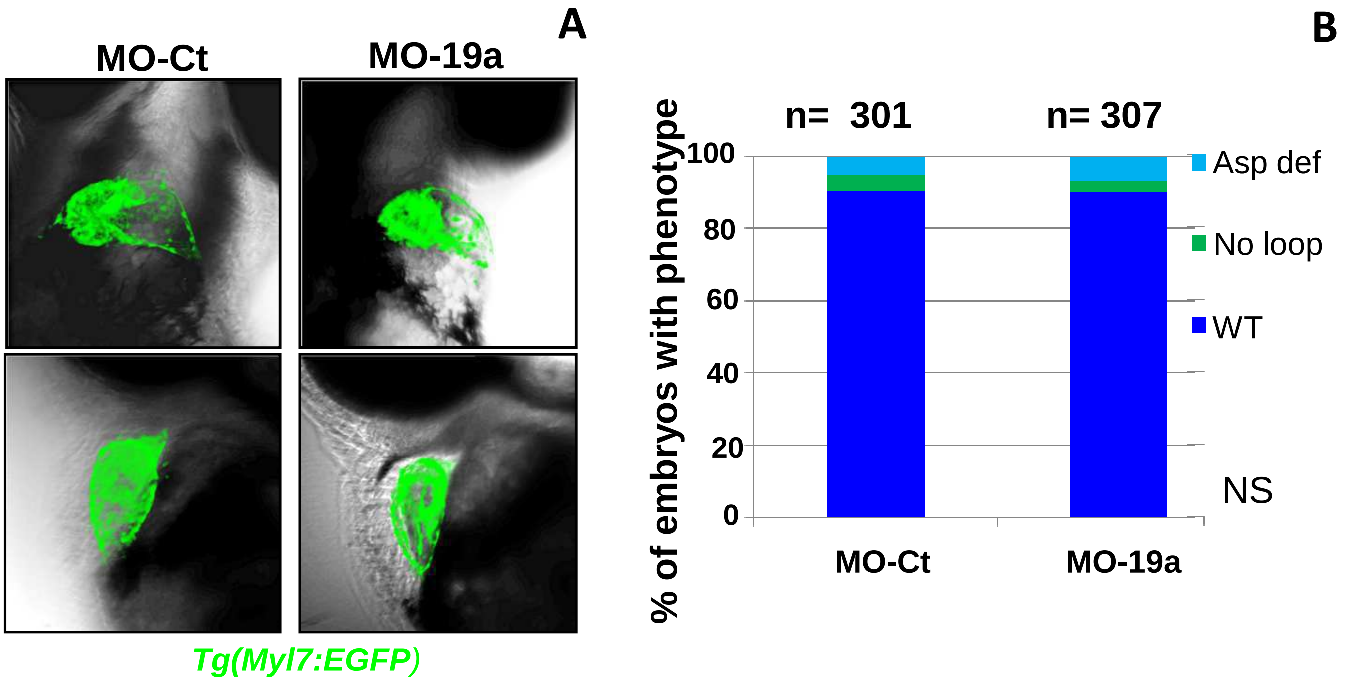

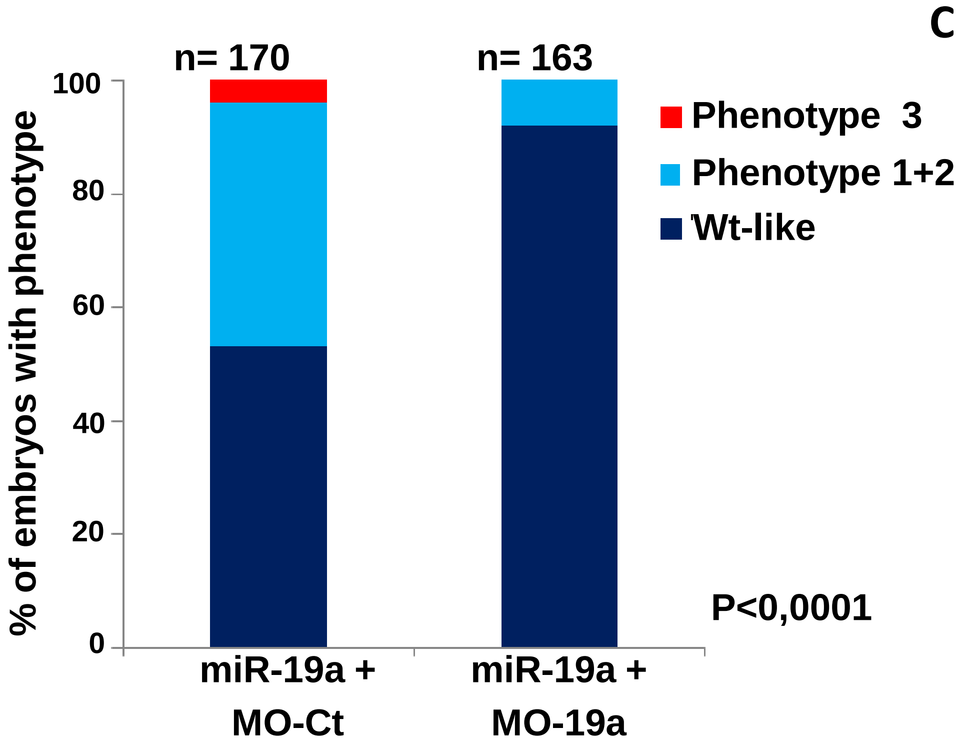

Figure S4. Co-injection of MO-19a is able to rescue cardiac defects induced by miR-19a over-expression.

Strain: Tg(*Tie2:EGFP*) +/-

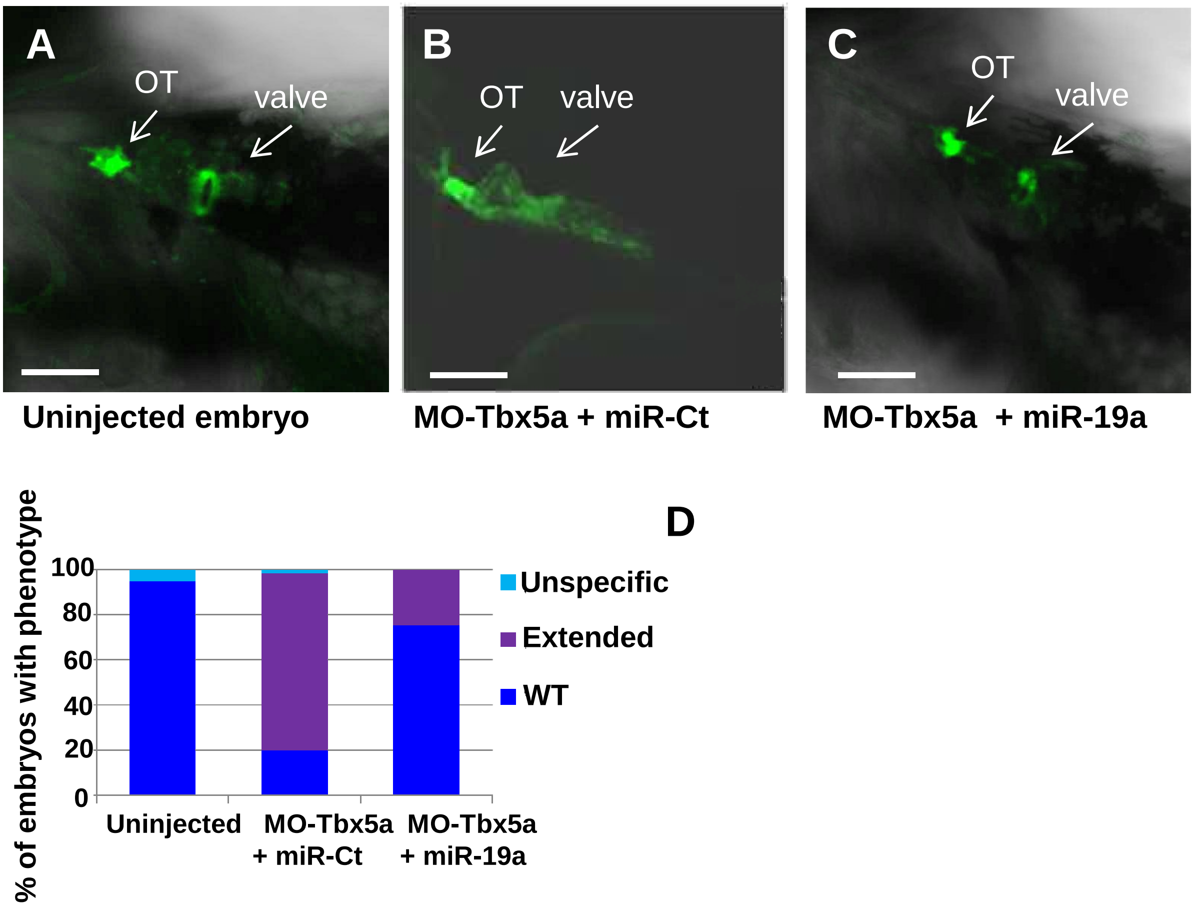

Figure S5. MiR-19a is able to rescue valve morphology.

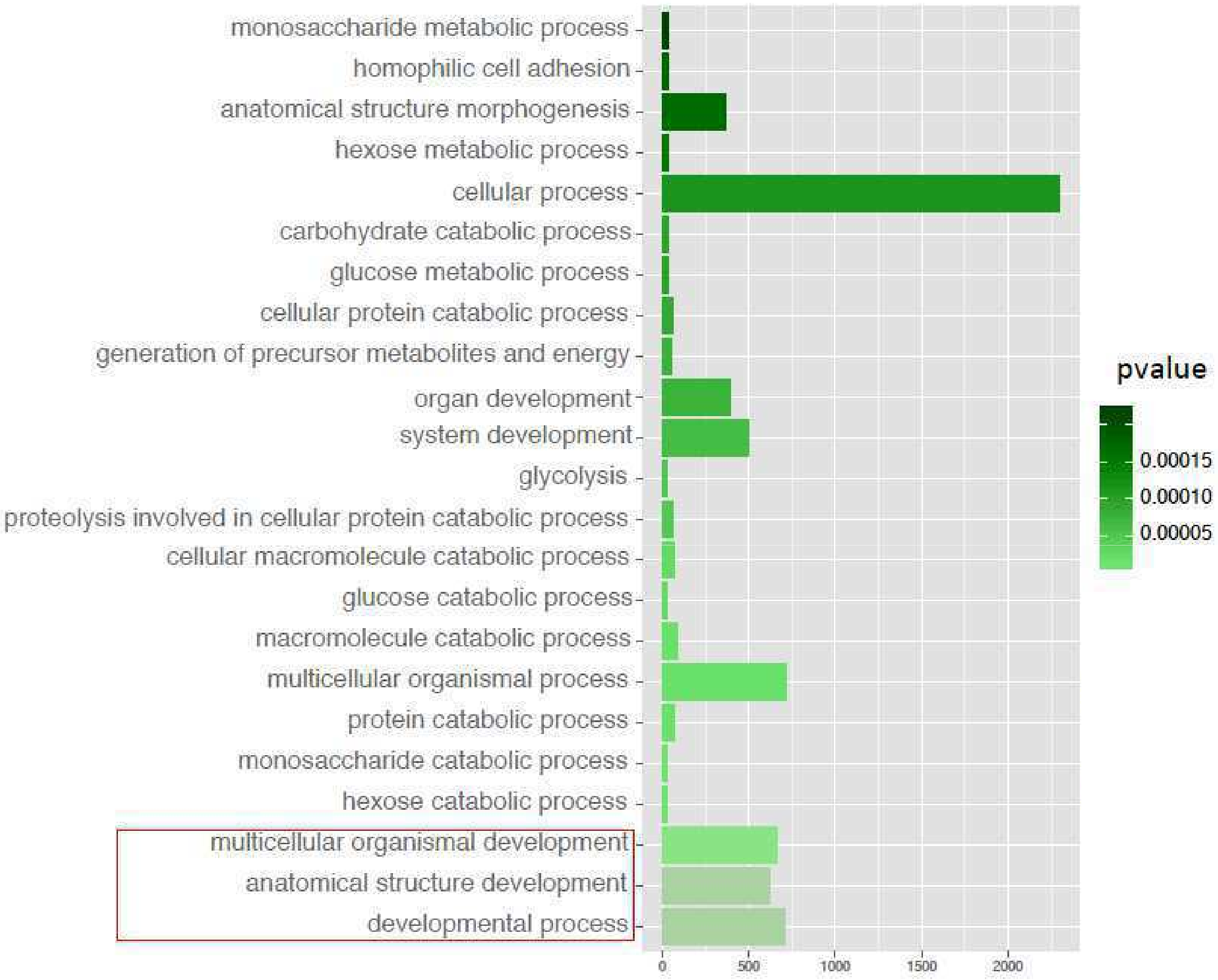

Figure S6. Development GO categories are overrepresented among the most influencing genes through first component of PCA analysis

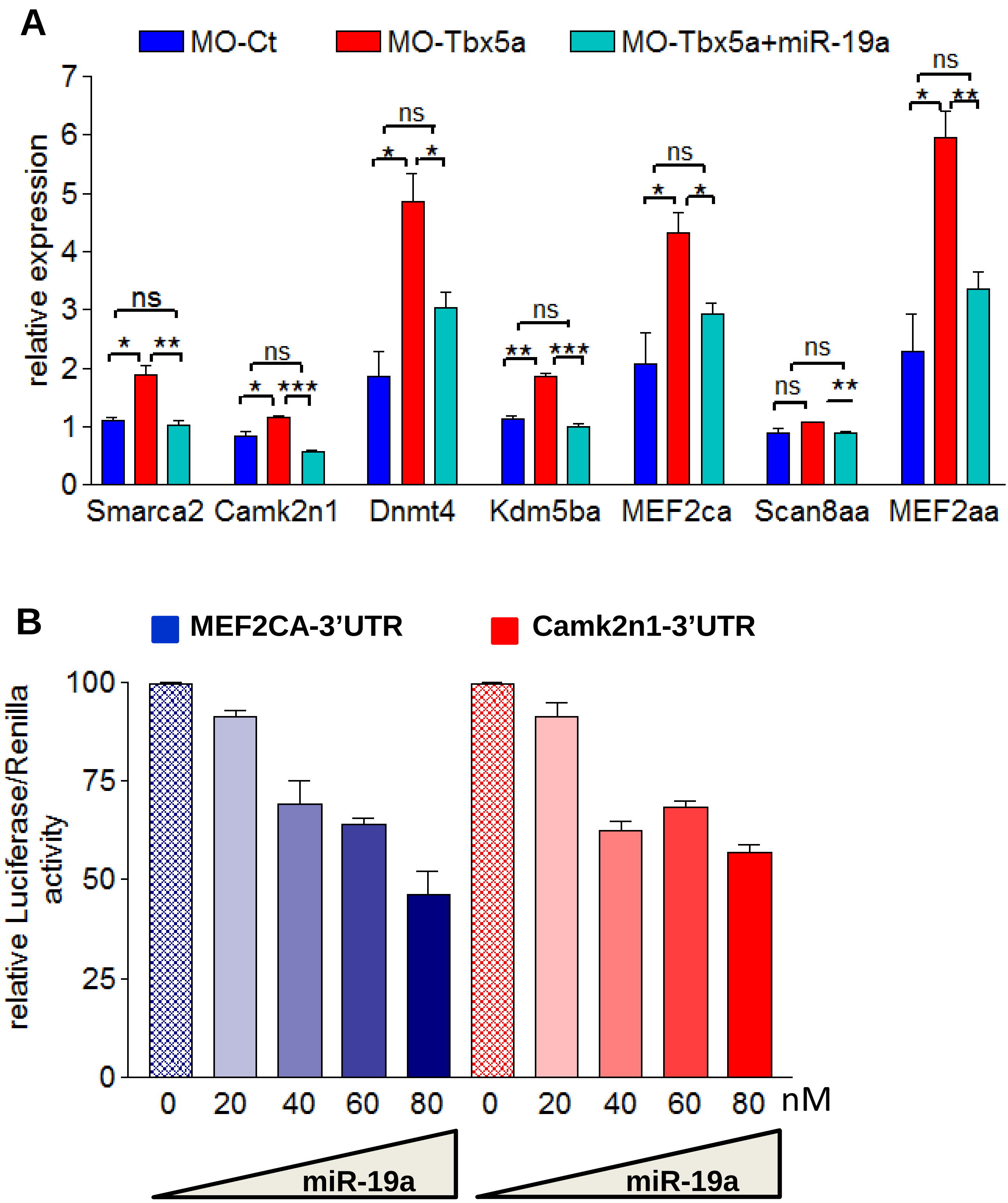

Figure S7. miR-19a target validation
